# Supplementary material for: Teaching the Evaluation of Female Pelvic Pain: A Hands-On Simulation to Reinforce Exam Skills and Introduce Transvaginal Ultrasound
Source: MedEdPORTAL. 2021 Jan 25;17:11080. doi: 10.15766/mep_2374-8265.11080 (PMC7830760; doi:10.15766/mep_2374-8265.11080)
Supplement: Supplementary file 1 — Simulation Case.docxDebrief PowerPoint.pptxFaculty Critical Action Checklist.docxStudent Survey.docx [file mep_2374-8265.11080-s001.zip › A. Simulation Case.docx]

| **Appendix A: Simulation Case**  SIMULATION CASE TITLE: Teaching the Evaluation of Female Pelvic Pain: A Hands-on Simulation Reinforcing Exam Skills and Introducing Transvaginal Ultrasound  AUTHORS: : Jennifer Pearson, MD,^1^ Amy Greminger, MD,^1^ Emily Onello, MD,^1^ Sandy Stover, MD^1^  ^1^Assistant Professor in the Department of Family Medicine and Biobehavioral Health, University of Minnesota Duluth campus  **LEARNER AUDIENCE:** Second year medical students | |
| --- | --- |
| **PATIENT NAME:** Renee  **PATIENT AGE:** 23  **CHIEF COMPLAINT:** abdominal and pelvic pain  **PHYSICAL SETTING:** Simulation Center: simulating the Emergency Department | |
|  | |
| **Brief narrative description of case** | The patient is a 23 year old female who presents to the Emergency Department with abdominal pain. Her history and physical exam are consistent with a diagnosis of pelvic inflammatory disease. Due to the patient’s hypotension and level of pain, the clinical concern for a complicating factor warrants further evaluation by use of a pelvic ultrasound. A bedside transvaginal ultrasound performed reveals a right sided tubo-ovarian abscess. Learner goals include clinical evaluation, discussion, and management of the presenting patient. |
| **Primary Learning Objectives** | 1. Demonstrate the ability to elicit a patient history given the presentation of female pelvic pain  2. Demonstrate empathy for a patient suffering from a painful condition such as pelvic inflammatory disease  3. Assess female patient with a targeted abdominal and pelvic exam through performance or observation  4. Develop a differential diagnosis for the presenting history and physical findings  5. Discuss the utility of transvaginal ultrasound in the evaluation of female pelvic pain  6. Demonstrate knowledge of pathophysiology, risk factors, clinical presentation, testing, diagnosis, management, reporting, and complications of pelvic inflammatory disease and tubo-ovarian abscess |
| **Clinical Actions** | 1. Take an appropriate medical history for presenting patient 2. Create an appropriate differential diagnosis for the presenting problem 3. Perform an appropriate physical exam including a thorough abdominal and female pelvic exam 4. Obtain appropriate laboratory sampling for suspected sexual transmitted infection 5. Perform a transvaginal ultrasound on patient |
| **Learner Preparation or Prework** | No specific pre-work is done prior to doing this simulation case. The case, however, should be situated within the curriculum at a point where students have already been taught basic skills in performing both the abdominal exam as well as female pelvic exam. Some familiarity with ultrasound basics is also recommended.  In addition, general knowledge of the disease states that can cause abdominal and pelvic pain will benefit students as they approach the simulation case and attempt to generate a differential diagnosis as well as narrow the diagnostic considerations. |

| Initial Presentation | | | |
| --- | --- | --- | --- |
| **Initial vital signs** | Blood Pressure: 90/50; Pulse: 120; Respirations: 20; O2 saturation: 98% on room air; Temperature: 103 F orally; Weight: 150 pounds | | |
| **Overall Setting and Appearance** | Students enter the simulation and see a mannequin dressed as a female, lying in an Emergency Department bed. | | |
| **Standardized participants and their roles in the room at case start** | Simulation Technician is trained to respond appropriately to specific abdominal and pelvic history questions and physical exam maneuvers. | | |
| **HPI** | *The following HPI is given to the medical students on the monitor at the onset of the case:*  Renee is a 23 year old female G1P1 brought to the Emergency Department complaining of abdominal pain that she rates as an 8 (on 1-10 scale). She was brought in by her boyfriend after she became dizzy in the shower and almost fainted. She has had the chills.  Renee has been experiencing some mild abdominal discomfort over the last week or so. She describes this as a nuisance, and as a 2-3 out of 10 on the pain scale. It has not affected her ability to do her daily activities, and so she just hadn’t come in for evaluation, hoping it would go away. When asked, she also states an increase in vaginal discharge over the last couple of weeks as well as dyspareunia over the last week or two.  *The following are the responses that the Simulation Technician would give to the student history questions:*  *Where is the pain located?* Lower abdomen, worse on the right side.  *When did it start?* Over the last week or two… has been more of a nuisance (about a 2 on the pain scale) and has not been interfering with activities of daily living, so she was just ignoring it, hoping it would go away.  *What is the course? Is it getting better or worse?* It is getting worse, especially since this morning.  *Quality?* Constant, dull, 8/10, worse with movement.  *When was the last menstrual period?* 3 weeks ago. Normal. Periods are regular.  *What is used for contraception?* Vaginal ring  *Are condoms used for STI protection?* No.  *Any injury?* No.  *Any similar pain in the past?* No.  *Any surgeries on the abdomen?* No.  *Any history of STI?* Yes. Genital herpes beginning 2 years ago, with two outbreaks, most recent 6 months ago.  *Last screening for STI?* At last annual exam 1 year ago.  *How long with current sexual partner?* 6 months.  *Partner with any symptoms?* No.  *Any vaginal discharge?* Yes, for two weeks.  *What color?* White/yellow.  *Odor?* Yes.  *Any pain with urination?* No.  *Any blood in the urine?* No.  *Any fevers or chills or sweats?* Yes, occasional chills and night sweats the past few nights.  *Any diarrhea?* Loose stools for 2-3 days.  *Any blood in stool?* No.  *Any Nausea or vomiting?* No vomiting. Yes, nausea today.  *Pain with intercourse/dyspareunia?* Yes, and over the last week to two this pain is getting worse.  *The following are further Review of Systems responses if students ask:*  *General:*  No weight change. Has been fatigued with general malaise past week or so. Also, with occasional chills and night sweats over the last few days. No actual temperature taken over this time (thermometer is broken and has not yet purchased another).  *Skin:*  No rashes, bruising, skin discoloration or changes.  *Head:*  Occasional migraine headaches (see Past Medical History) No. trauma, dizziness or seizures.  *Eyes:*  No visual field deficits, discomfort, glaucoma, diplopia.  *Ears:*  No tinnitus, vertigo, hearing loss, drainage or discharge.  *Nose:*  No epistaxis, sinus infections, congestion, discharge.  *Mouth and Throat:*  No oral sores, dentition, hoarseness, throat pain, masses, dysphagia.    *Neck:*  No masses, goiter, pain, range of motion.    *Breasts:*  No lumps, discharge, pain, tenderness, self-exam.    *CV:*  No chest pain, orthopnea, PND, dyspnea on exertion, palpitation, claudication.  *Respiratory:*  No cough, shortness of breath, wheezing, sputum, hemoptysis.  *GI:*  Has had abdominal pain (see History of Present Illness). Stools loose over the last 2-3 days. No melena, hematochezia, hematemesis, dysphagia. Currently nauseous. No vomiting.    *GU:* No dysuria, frequency, nocturia, urgency, incontinence, hesitancy, hematuria, or double voiding.  *GYN:* Has had vaginal discharge, dyspareunia (see History of Present Illness). No recent herpes outbreaks or prodromal syndromes. No dysmenorrhea or vaginal bleeding. No orgasmic problems.  *Endocrine:*  No polyuria, polyphagia, polydipsia, skin or hair changes, heat or cold intolerance.    *Musculoskeletal:* No pain, swelling or problems with range of motion.  *Heme/Lymph:*  No bleeding disorders, easy bruising, or lymphadenopathy.  *Neuro/Psych:*  Reports no weakness, seizures, gait imbalance, memory changes, anxiety. Depression controlled on Effexor. No reported change in mental status. | | |
| **Past Medical/Surgical History** | **Medications** | **Allergies** | **Family History** |
| 1. G1P1, History of vaginal delivery of her son 3 years ago. Term pregnancy, no complications with pregnancy, labor or delivery. Delivered at Essentia Health with epidural anesthesia. 2. History of migraine headaches. 3. History of depression, well controlled. 4. History of genital herpes. First outbreak 2 years ago, has had two outbreaks since, last one 6 months ago.   Social History: Had been married for 3 years. Divorced 1 ½ years ago due to husband’s infidelity. Mother and one sister in town. Living with current boyfriend for 6 months. Primarily a stay at home mom with 3 year old son, Jack, but looking to get a part time job as there are many financial concerns. Boyfriend works at a call center. Graduated from high school and one year of community college. She enjoys attending playgroups with her son.  Habits: Tobacco use: smokes occasionally while out drinking with friends. ETOH: drinks with friends on weekends, up to 4-5 beers/night on those evenings. No other drug use. Exercise 3 times per week, walking for about 30 minutes. Diet is standard American diet. Sleep: no problems. | Effexor XR 75 mg/day  Fioricet 1-2 tabs q 4 hours PRN migraine headache  Nuvaring  Valtrex PRN herpes outbreaks | Sulfa-rash | Mother with breast cancer age 56- still living.  Father with hypertension. No other family history cancer, diabetes, TB, hypertension, bleeding disorders, heart disease. |
| **Physical Examination** | | | |
| **General** | Uncomfortable appearing, lying very still, shivering. | | |
| **HEENT** | Unremarkable. Normal findings. | | |
| **Neck** | Unremarkable. Normal findings. | | |
| **Lungs** | Lungs clear throughout anterior and posterior lung fields. | | |
| **Cardiovascular** | Regular S1S2 without murmur, rub or gallop appreciated. | | |
| **Abdomen** | - Occasional bowel sounds. - Inspection of abdomen is normal without visible distention or mass. - Right lower quadrant (RLQ) with guarding and rebound tenderness. - Significant tenderness to deep palpation throughout RLQ. - No abnormal findings to abdominal percussion beyond patient discomfort while percussing over RLQ. - Rovsing’s negative. - Psoas and obturator signs positive on right, negative on left. - Murphy’s negative. | | |
| **Neurological** | Unremarkable. Normal neurologic exam with no focal findings. | | |
| **Skin** | Unremarkable. No rashes or other dermatologic findings. | | |
| **Female Pelvic Exam** | - Normal appearing external genitalia without lesions. - Speculum exam reveals friable, tender cervix. Mild to moderate whitish, purulent appearing discharge present in posterior fornix and at external cervical os. - Bimanual exam reveals significant cervical motion tenderness. Uterus normal sized. Significant tenderness to palpation over right adnexa. Difficult to get a good exam due to amount of guarding and tenderness present, but exam reveals suspicion of an adnexal mass on the right. Left sided adnexal exam without mass appreciated, but mild to moderate tenderness to palpation. - Rectovaginal exam reveals tenderness on the right side greater than left. | | |
| **Psychiatric** | No specific findings. Patient is appropriate in her responses throughout exam. | | |
| **Labs** | - Complete Blood Count: WBC 17,000, Hgb 12.5, Plt 318 - Differential shows left shift - Urine pregnancy test: negative - Serum HCG: negative - Urinalysis: Moderate epithelial cells, 15-20WBC/hpf, 2-5 RBC/hpf, Few bacteria. Dip is negative for ketones, glucose, protein, and nitrates, but positive for blood, leuk esterase - C-Reactive Protein is 7.2 mg/dL (normal= less than 0.8 mg/dL) - Sedimentation rate is elevated at 30 mm/hr (normal 0-20 mm/hr) - Blood cultures are obtained and will be available in 24-48 hours - Swab for Gonorrhea/Chlamydia is obtained and will be available in 24-48 hours - Wet prep shows PMN’s and bacteria. No yeast or trichomonas - Ultrasound of pelvis shows: right sided tubo-ovarian abscess (students must perform this with use of bedside transvaginal ultrasound) | | |

| Instructor Notes – Changes and CASE Branch Points | | |
| --- | --- | --- |
| **Intervention / Time point** | **Change in Case** | **Additional Information** |
| Approximately 5-10 minutes into the case when students are performing an abdominal exam |  | Simulation Technician is taught how to respond appropriately to specific abdominal exam techniques i.e.:  When students palpate the right lower quadrant, patient states “ouch, that hurts a lot!”  Specific patient response to each exam technique is scripted. |
| Approximately 10-15 minutes into the case, when students consider need to do a pelvic exam | Faculty enters and talks students through the appropriate draping and technique in performing a female pelvic exam, raising questions about what is seen and felt throughout exam (i.e.: purulent cervicitis, cervical motion tenderness, significant pain with attempt to palpate right adnexa), as well as what sampling might be desired (i.e. inappropriate to do PAP in this context, consider wet prep, STI testing). | Vaginal ring had been inserted into vaginal vault of pelvic model to be consistent with case. This may be pointed out to student. Pelvic model had also been fitted with cervix that had purulent appearing discharge present at external os.  In addition, faculty let student performing the exam know how patient would respond. For example:  During bimanual exam when student inserts fingers into vaginal vault and palpates cervix, faculty would indicate that this would be extremely painful and not tolerated well by patient. |
| Following appropriate pelvic exam and sampling, when students are considering a differential diagnosis | Labs are ordered | Results are given to students after a short delay |
| Approximately 15-20 minutes into the case, when students consider the need for a pelvic ultrasound | Faculty again enters the simulation scenario and suggests that students perform an ultrasound with an available bedside machine. The small group then moves to the female pelvic ultrasound model, where the faculty guides students in practice of transvaginal ultrasound technique. The normal anatomy is reviewed, and the ovarian pathology is discovered. Faculty allows students to consider the possible differential diagnosis of what is discovered on ultrasound (a right ovarian mass consistent with tubo-ovarian abscess) as well as what they would consider for management given this complication. | Given the level of pain that the patient may experience with a transvaginal probe within the clinical scenario, faculty also discusses with students the likely use of abdominal probe first to see if adequate visualization of pathology can be accomplished. Given the learning objective to begin development of skills with use of transvaginal probe, faculty guides conversation regarding situations where transvaginal probe may be needed (i.e.: high BMI with inadequate visualization of reproductive pathology with abdominal probe) as well as the need to use adequate pain medication before introduction of transvaginal probe into vaginal vault. |
| Following pelvic ultrasound | Students discuss their clinical evaluation, differential, and plan at this point in the simulation. | Simulation is stopped, with further discussion to be continued during the debrief. |

**Ideal Scenario Flow**

The medical students enter the simulation room and find the simulation mannequin dressed as a young woman lying in bed. She is appropriately conversive but communicates distress secondary to her level of abdominal/pelvic pain. Vitals and patient history are obtained. Physical exam is performed, with keyed response from Simulation Technician to indicate significant right lower quadrant pain. Students should recognize the need to include a pelvic exam as part of the evaluation of this patient scenario. Clinical faculty then steps into the simulation scenario and offers guidance for the pelvic exam. Students move to the pelvic model stationed right next to simulation mannequin and perform a pelvic exam along with the appropriate sampling for possible STI’s (sampling guided by faculty). Faculty then steps out of the scenario and allows the students to discuss and develop differential diagnosis and decide on labs and further testing desired.

When students reach the point of the case where they are considering ordering an ultrasound, faculty again steps into scenario and offers guidance through a bedside ultrasound that is available (hidden from view from students until this point of the case, when ultrasound and a pelvic model with associated pathology are then uncovered). Students then move to the ultrasound and with the use of a transvaginal probe, are guided by faculty as to technique and visualization of both normal anatomy as well as underlying pathology. Students recognize right sided ovarian mass. Faculty prompts questions regarding what this could represent in the context of the case, and simulation is stopped with students left considering differential and final diagnosis.

The diagnosis of pelvic inflammatory disease with tubo-ovarian abscess is then reviewed with a PowerPoint slide presentation debrief following the scenario. Key teaching points as outlined on Faculty Critical Action Checklist (Appendix C) are discussed. Content reviewing pelvic inflammatory disease and tubo-ovarian abscess is also reviewed, along with appropriate management guidelines.

**Anticipated Management Mistakes**

*Provide a list of management errors or difficulties that are commonly encountered when using this simulation case.*

1. Uncertainty over how to perform a female pelvic exam: We found that faculty presence within the simulation experience allowed faculty to verbally guide students through an appropriate pelvic exam, with correct technique reiterated for all students in the small group.
2. Uncertainty over how to perform lab sampling for suspected sexually transmitted infection: We found that faculty presence during this portion of the simulation experience allowed for a facilitated discussion of what sampling students would consider, and how sampling should be performed.
3. Uncertainty over how to perform a pelvic ultrasound: Faculty guidance during this portion of the simulation allowed small group discussion and teaching regarding appropriate technique for use of transvaginal probe, recognition of normal female anatomy with ultrasound, and recognition of the pathology present.
4. Uncertainty about final diagnosis: Once right sided ovarian pathology was visualized by ultrasound, students were often unclear about the exact diagnosis. Faculty encouraged further consideration and discussion of the overall clinical scenario and the resulting differential diagnosis. Most of our learners were unfamiliar with the use of transvaginal probe for pelvic pathology evaluation, as well as how to interpret findings. Specific debriefing content was thus included in a slide set (Appendix B) to cover this clinical diagnosis and management information.
