# Supplementary material for: Teaching the Evaluation of Female Pelvic Pain: A Hands-On Simulation to Reinforce Exam Skills and Introduce Transvaginal Ultrasound
Source: MedEdPORTAL. 2021 Jan 25;17:11080. doi: 10.15766/mep_2374-8265.11080 (PMC7830760; doi:10.15766/mep_2374-8265.11080)
Supplement: Supplementary file 1 — Simulation Case.docxDebrief PowerPoint.pptxFaculty Critical Action Checklist.docxStudent Survey.docx [file mep_2374-8265.11080-s001.zip › B. Debrief PowerPoint.pptx]

## Slide 1
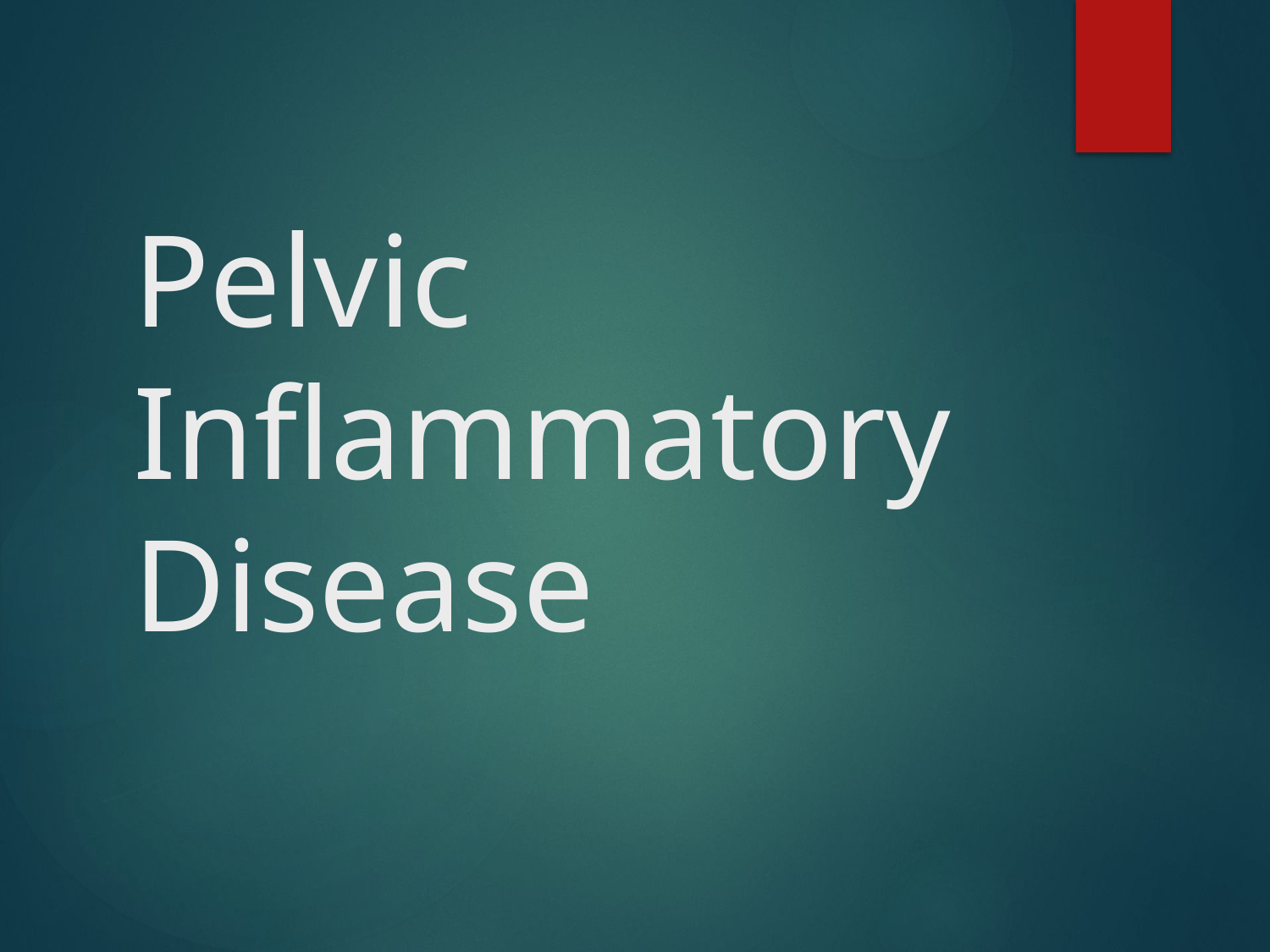

# Pelvic Inflammatory Disease

## Slide 2
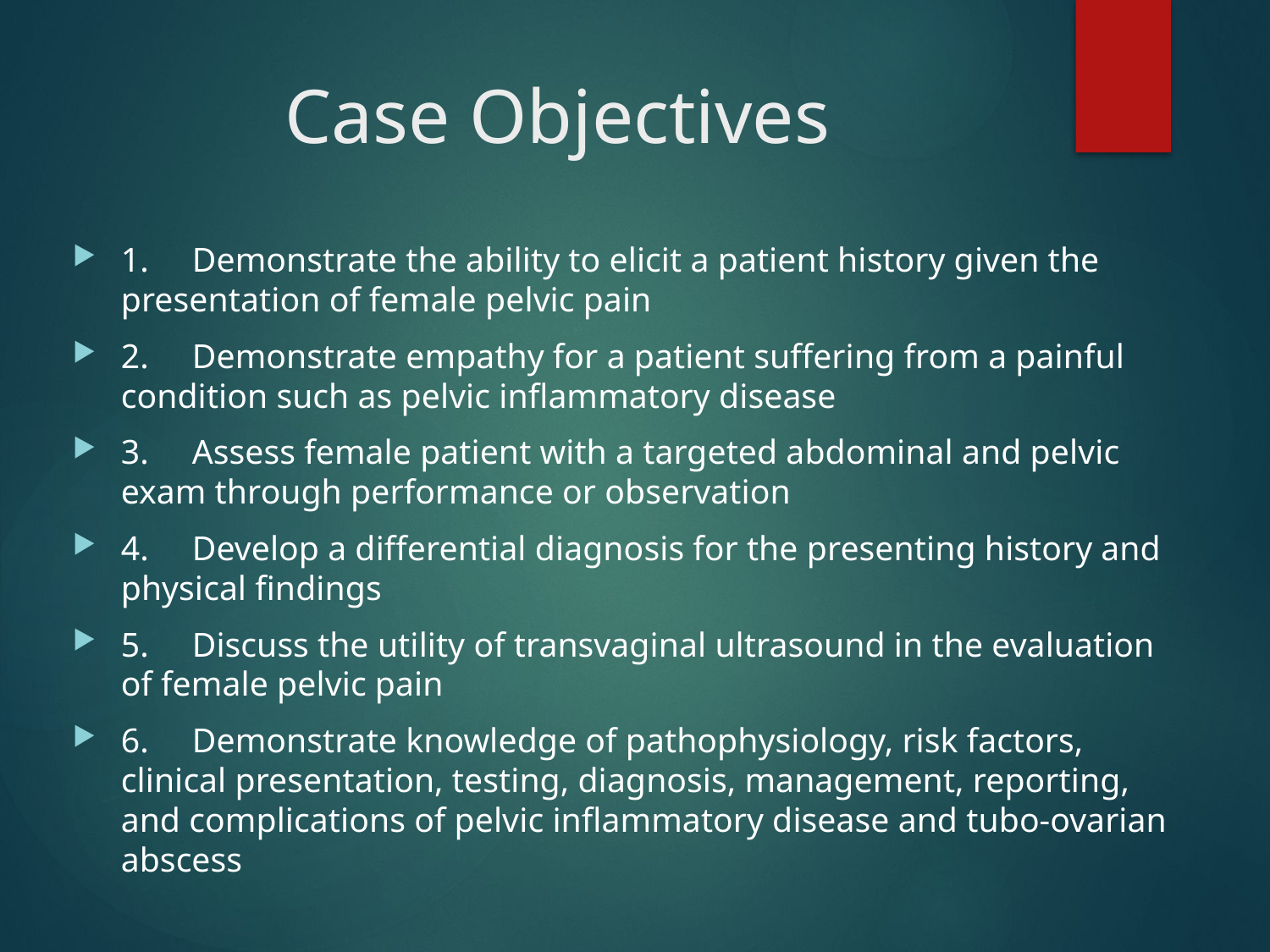

# Case Objectives
1. Demonstrate the ability to elicit a patient history given the presentation of female pelvic pain
2. Demonstrate empathy for a patient suffering from a painful condition such as pelvic inflammatory disease
3. Assess female patient with a targeted abdominal and pelvic exam through performance or observation
4. Develop a differential diagnosis for the presenting history and physical findings
5. Discuss the utility of transvaginal ultrasound in the evaluation of female pelvic pain
6. Demonstrate knowledge of pathophysiology, risk factors, clinical presentation, testing, diagnosis, management, reporting, and complications of pelvic inflammatory disease and tubo-ovarian abscess

## Slide 3
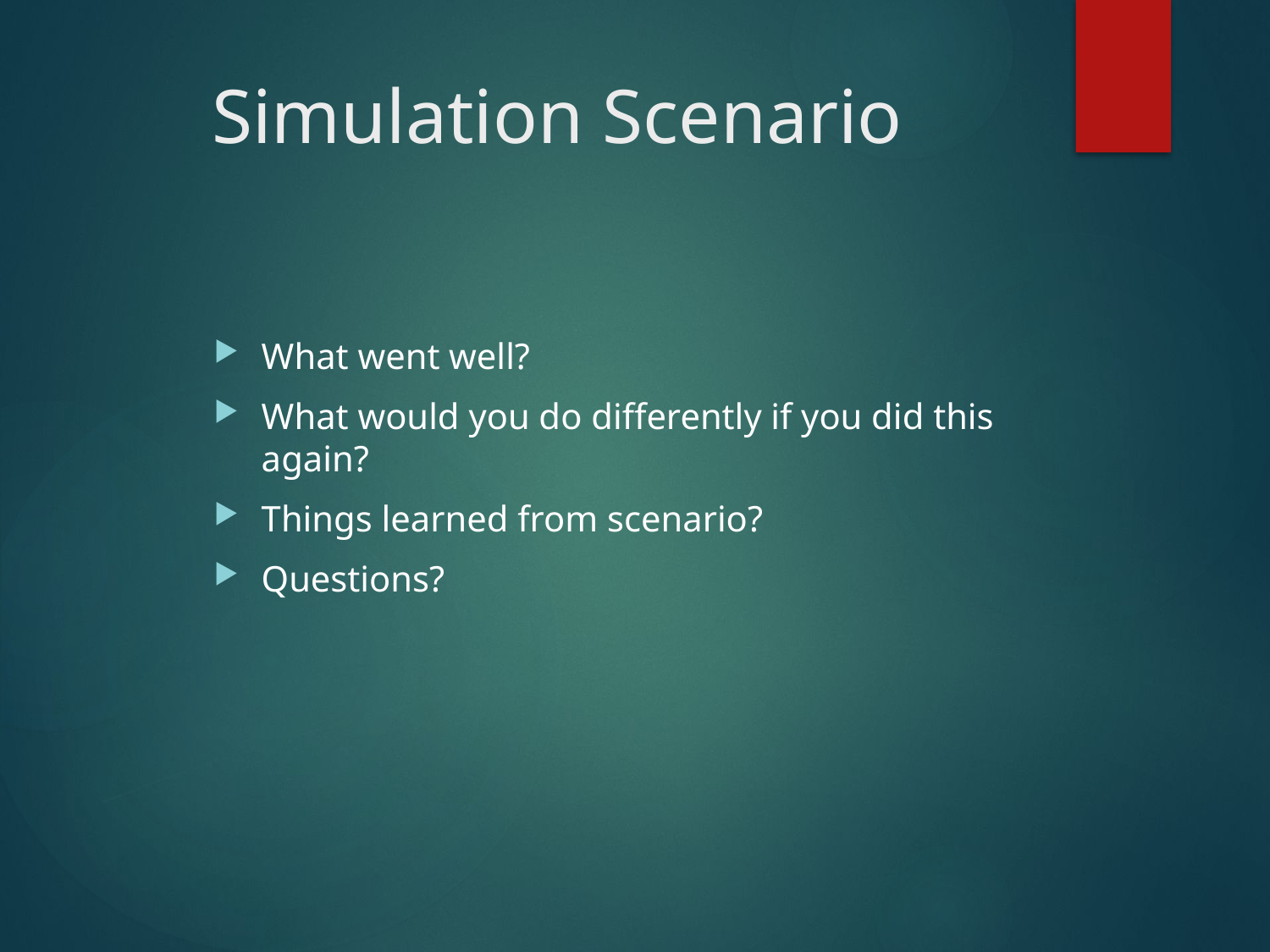

# Simulation Scenario
What went well?
What would you do differently if you did this again?
Things learned from scenario?
Questions?

## Slide 4
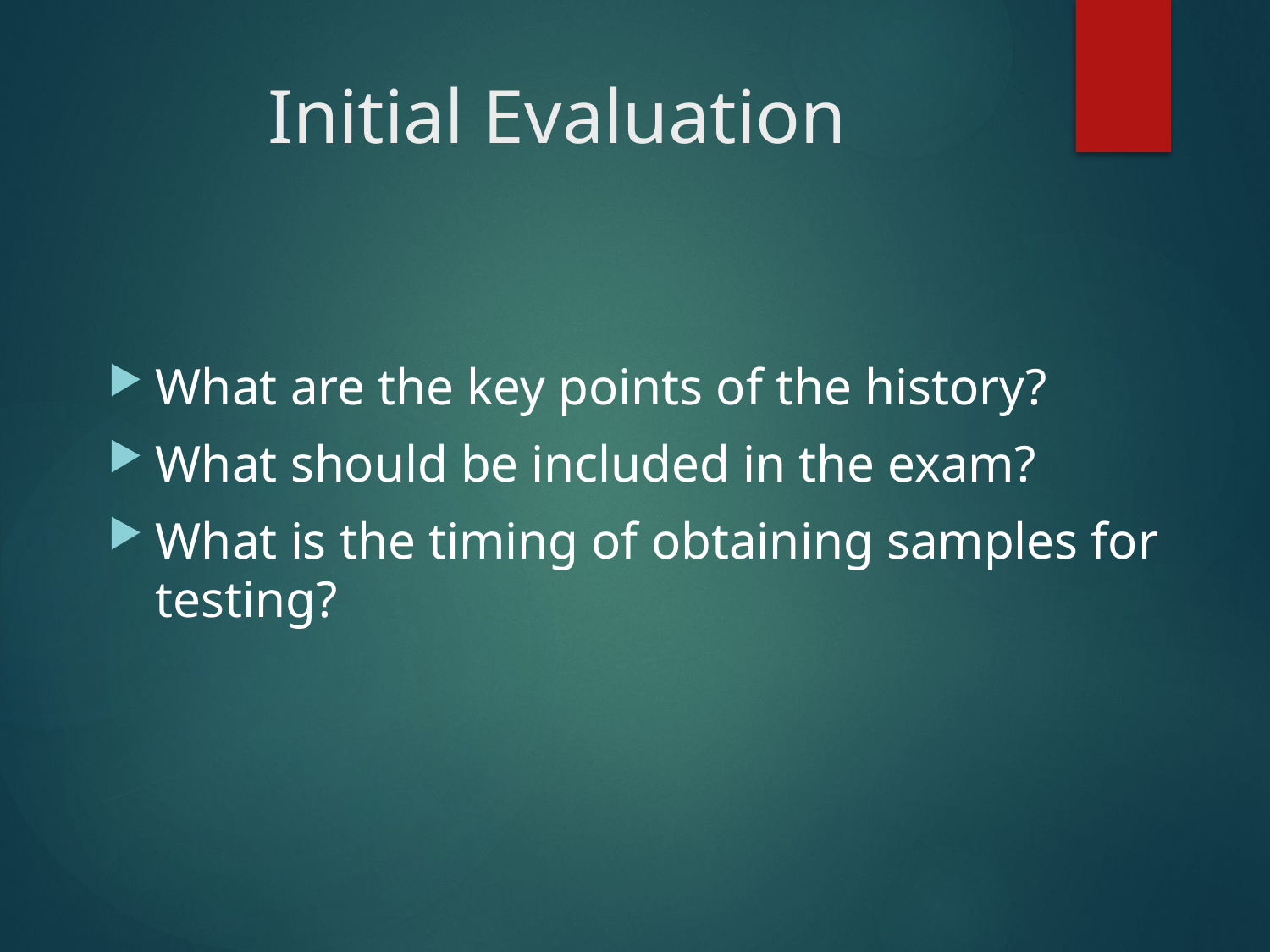

# Initial Evaluation
What are the key points of the history?
What should be included in the exam?
What is the timing of obtaining samples for testing?

## Slide 5
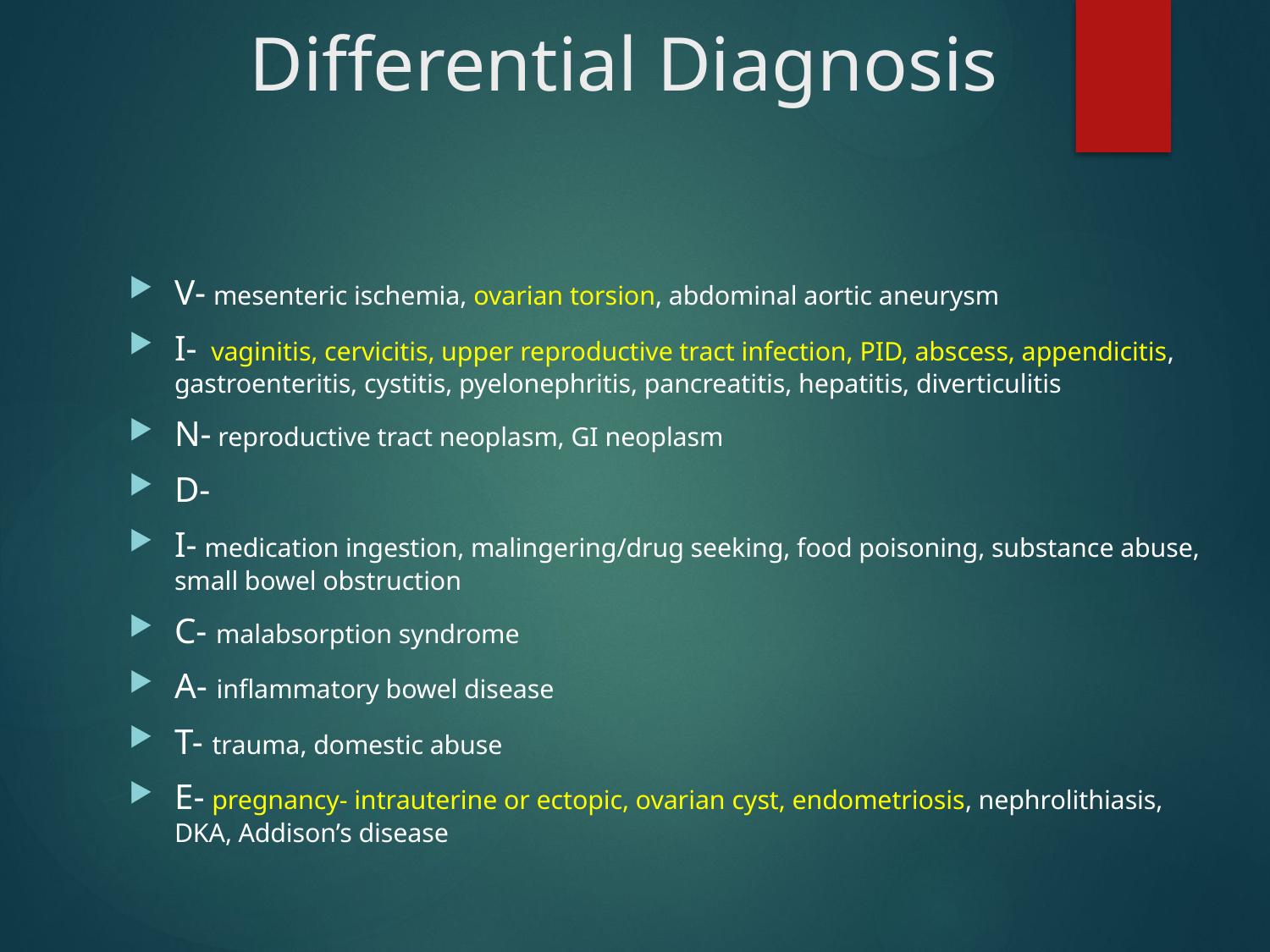

# Differential Diagnosis
V- mesenteric ischemia, ovarian torsion, abdominal aortic aneurysm
I- vaginitis, cervicitis, upper reproductive tract infection, PID, abscess, appendicitis, gastroenteritis, cystitis, pyelonephritis, pancreatitis, hepatitis, diverticulitis
N- reproductive tract neoplasm, GI neoplasm
D-
I- medication ingestion, malingering/drug seeking, food poisoning, substance abuse, small bowel obstruction
C- malabsorption syndrome
A- inflammatory bowel disease
T- trauma, domestic abuse
E- pregnancy- intrauterine or ectopic, ovarian cyst, endometriosis, nephrolithiasis, DKA, Addison’s disease

## Slide 6
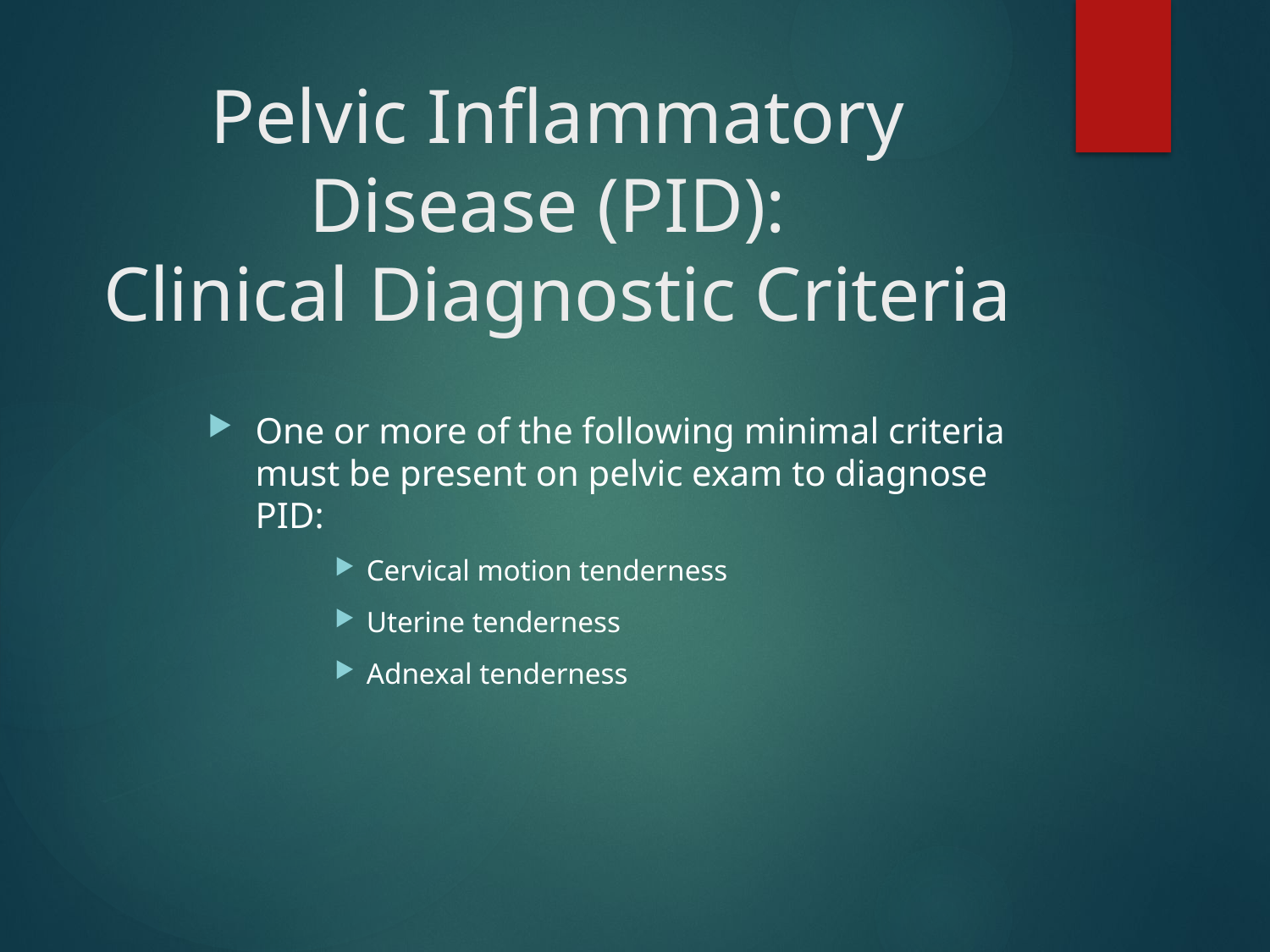

# Pelvic Inflammatory Disease (PID): Clinical Diagnostic Criteria
One or more of the following minimal criteria must be present on pelvic exam to diagnose PID:
Cervical motion tenderness
Uterine tenderness
Adnexal tenderness

## Slide 7
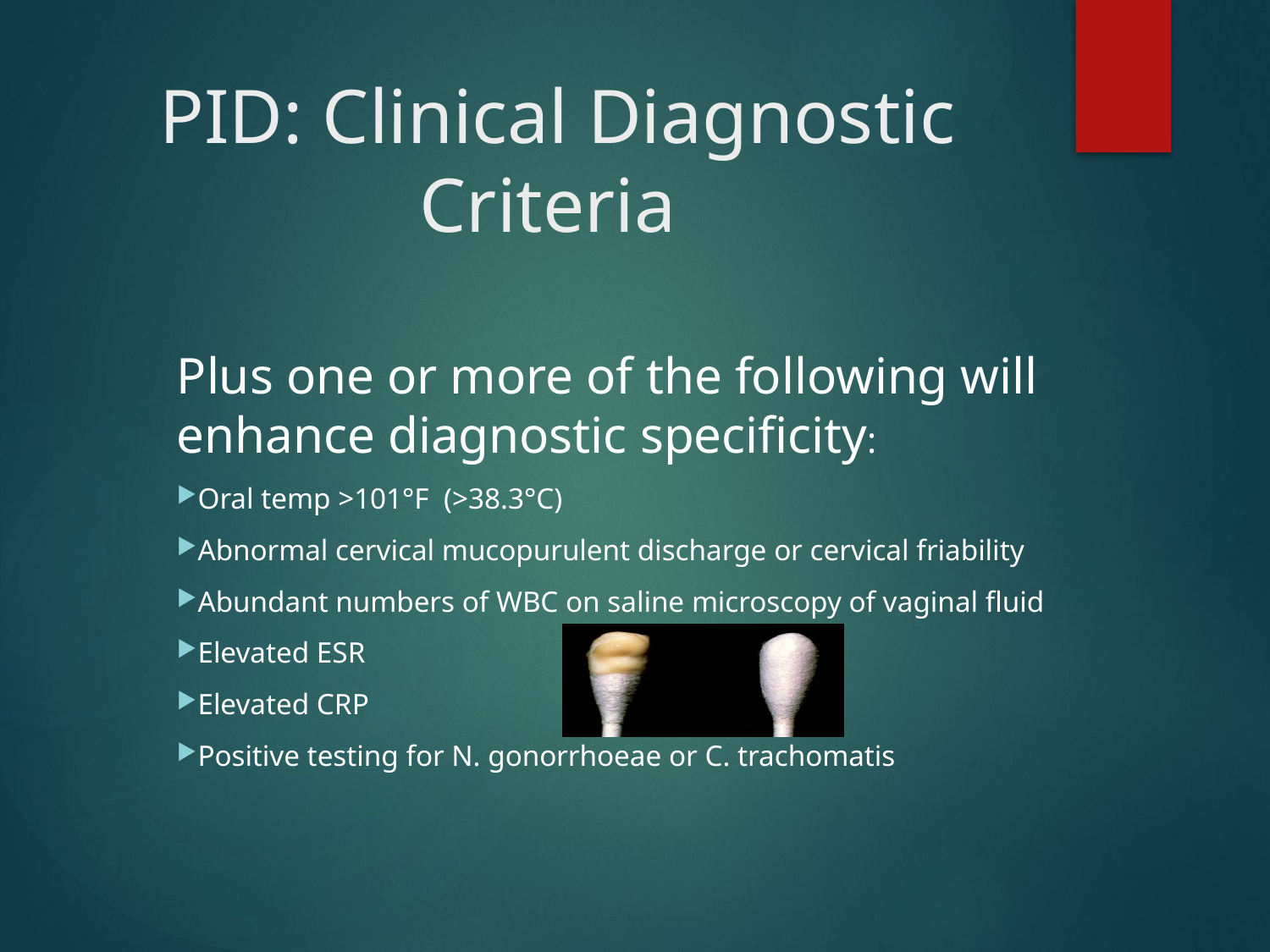

# PID: Clinical Diagnostic Criteria
Plus one or more of the following will enhance diagnostic specificity:
Oral temp >101°F (>38.3°C)
Abnormal cervical mucopurulent discharge or cervical friability
Abundant numbers of WBC on saline microscopy of vaginal fluid
Elevated ESR
Elevated CRP
Positive testing for N. gonorrhoeae or C. trachomatis

## Slide 8
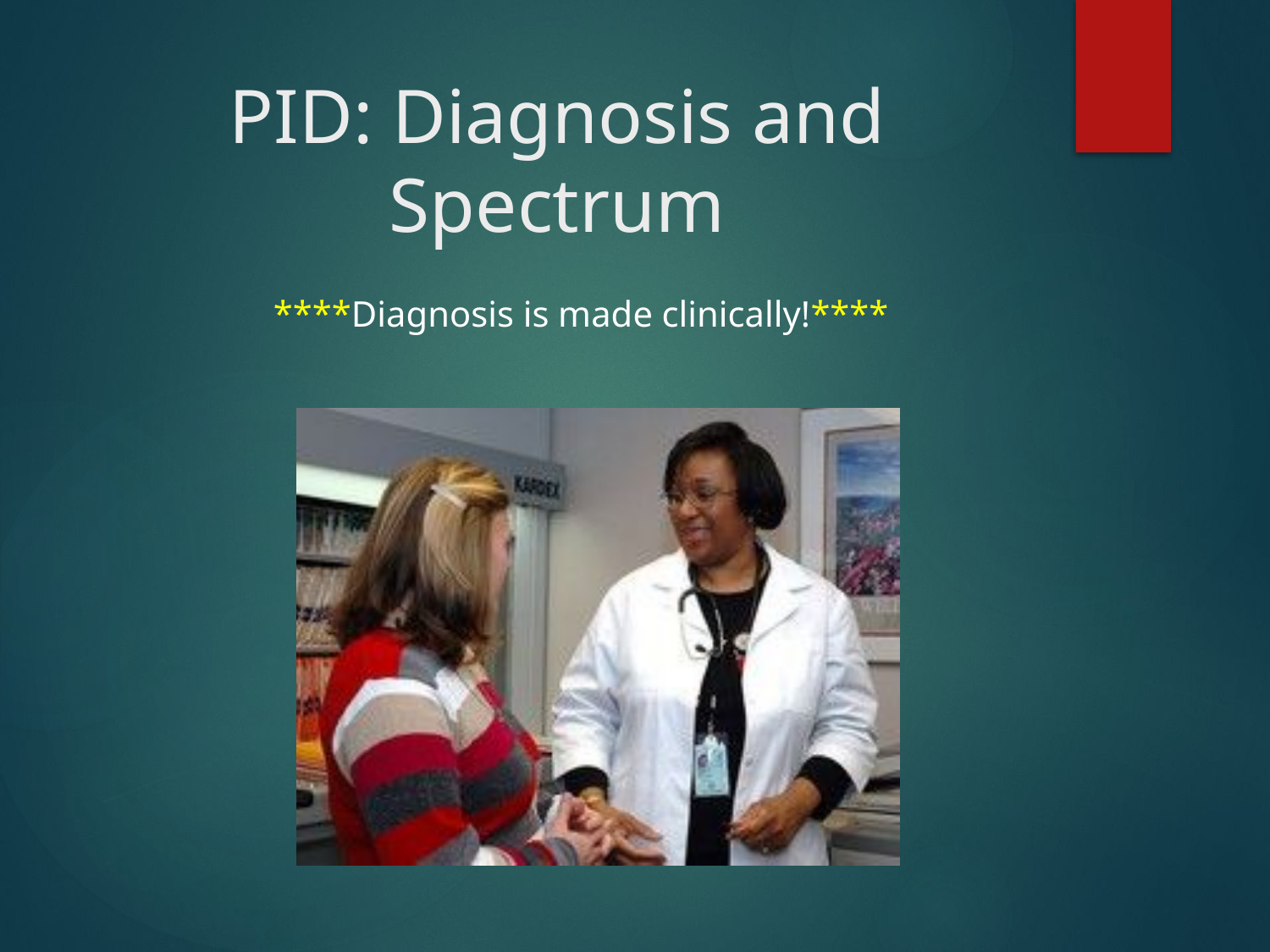

# PID: Diagnosis and Spectrum
****Diagnosis is made clinically!****

## Slide 9
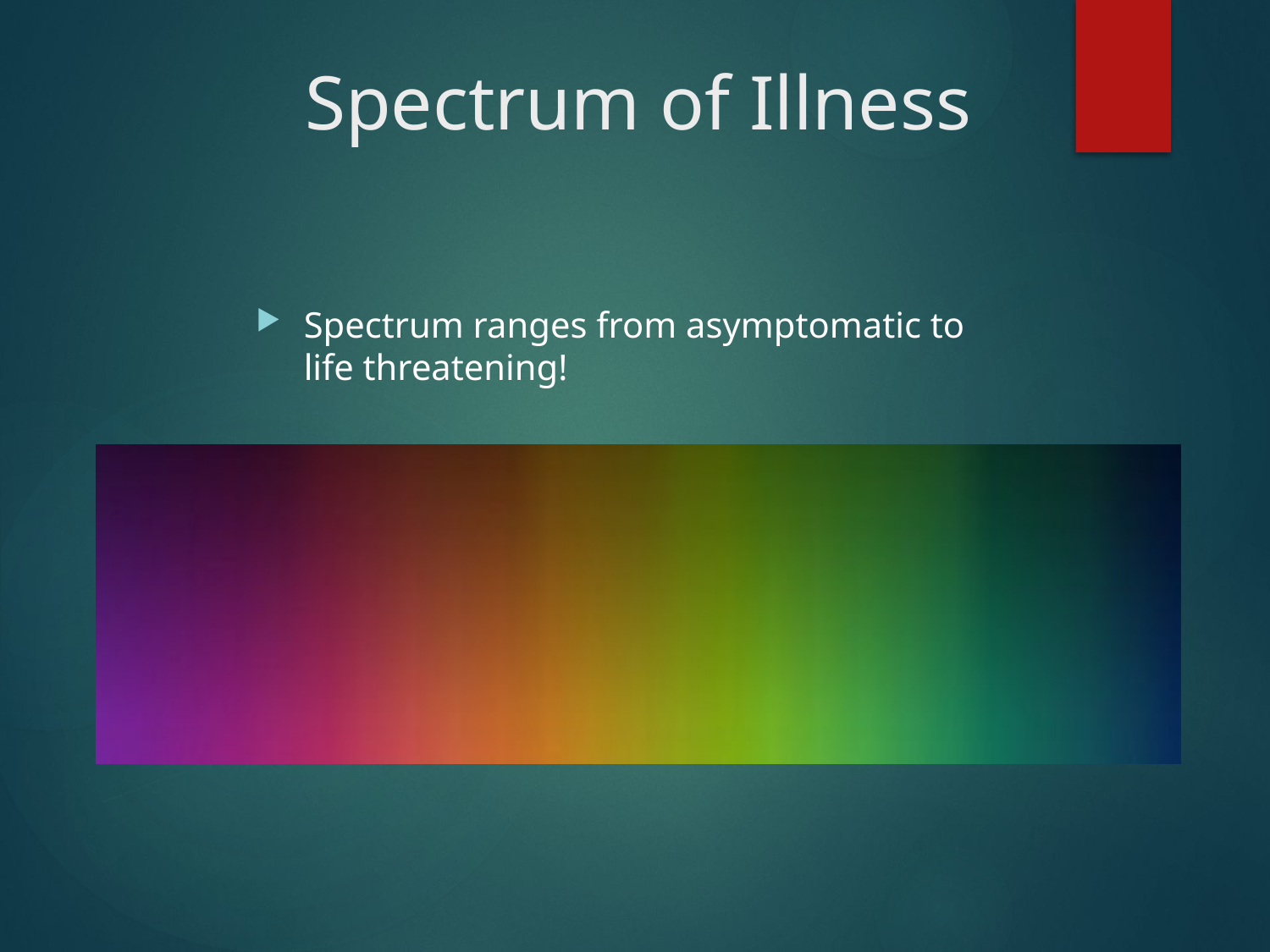

# Spectrum of Illness
Spectrum ranges from asymptomatic to life threatening!

## Slide 10
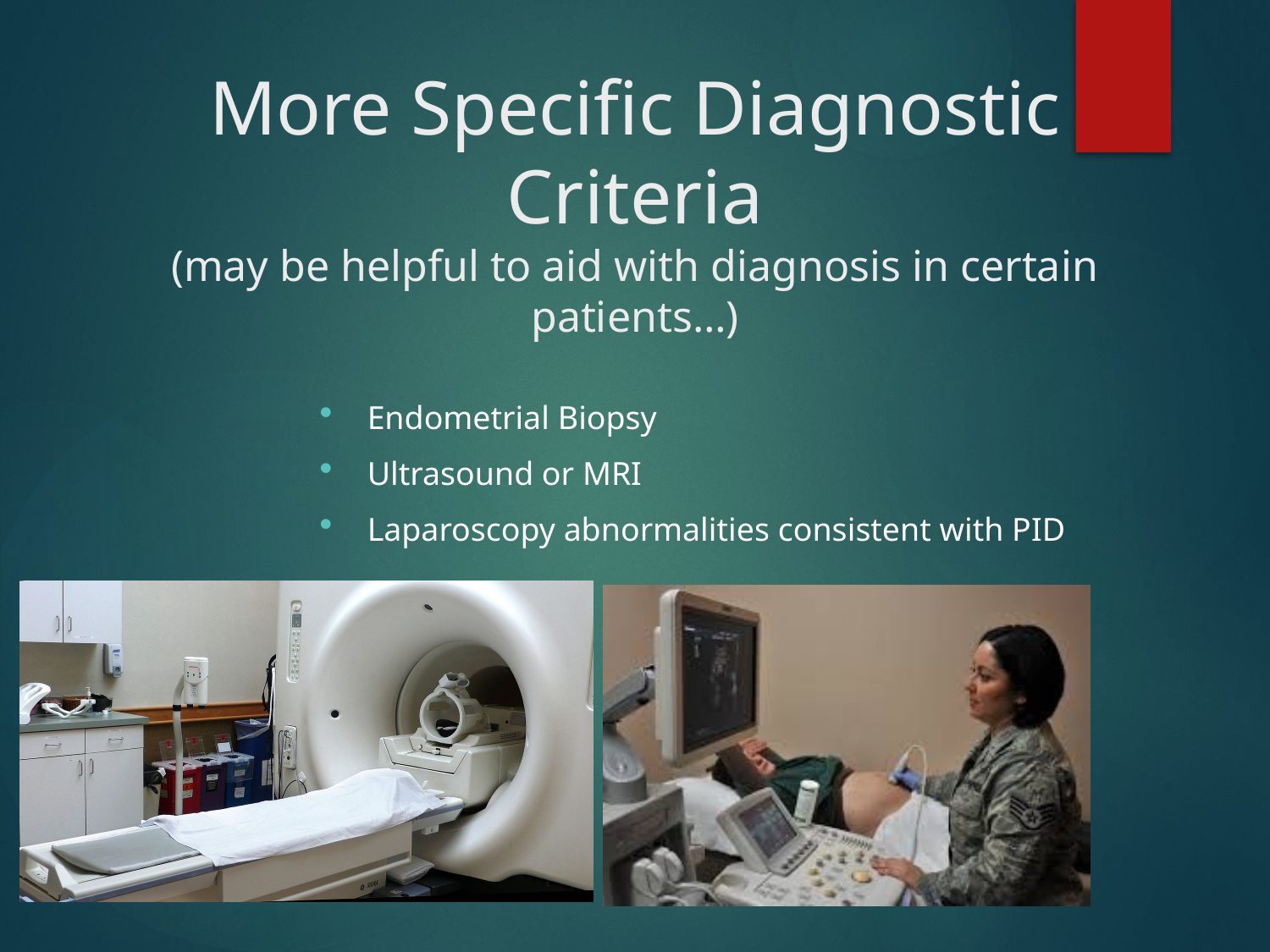

# More Specific Diagnostic Criteria (may be helpful to aid with diagnosis in certain patients…)
Endometrial Biopsy
Ultrasound or MRI
Laparoscopy abnormalities consistent with PID

## Slide 11
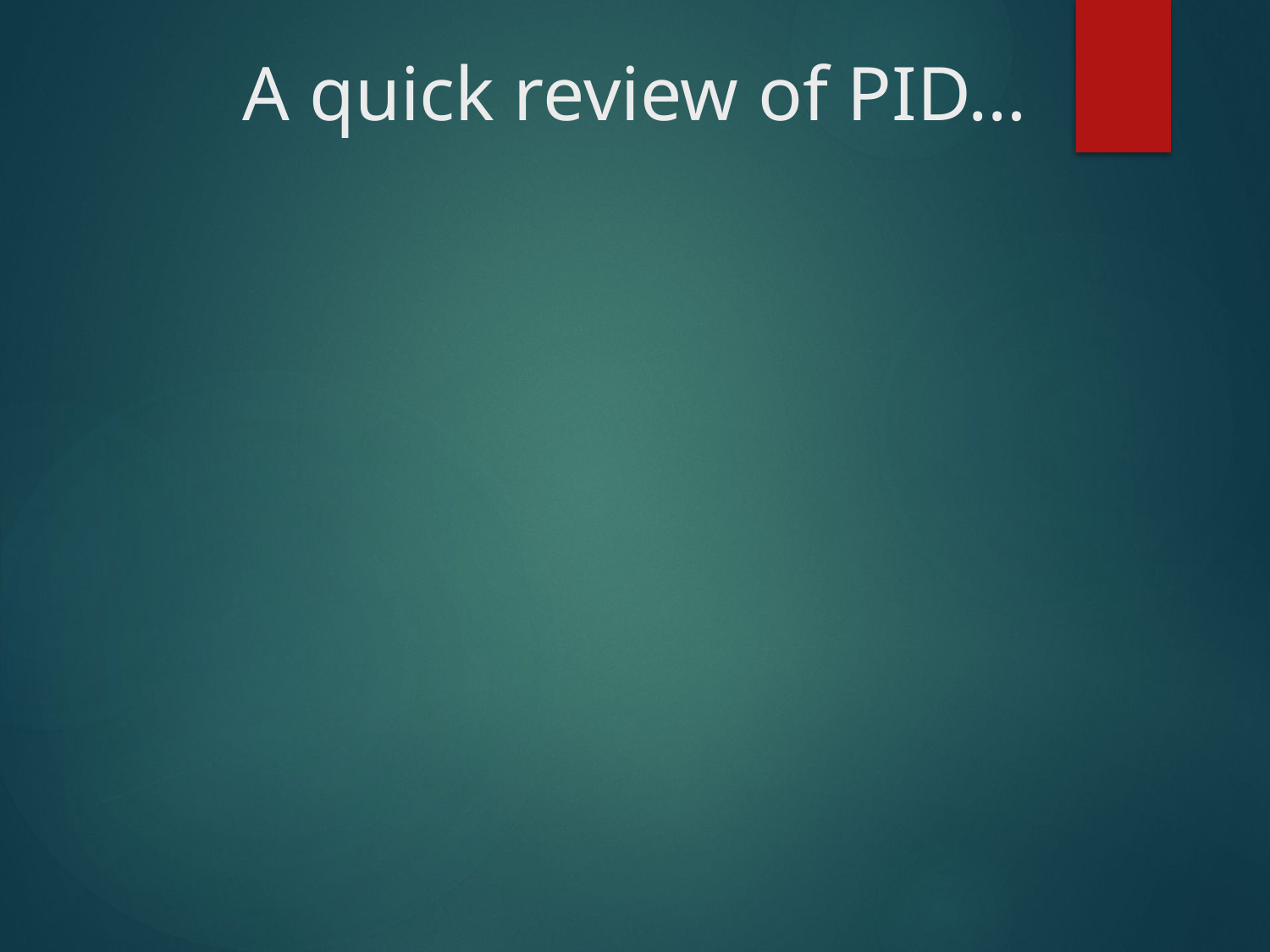

# A quick review of PID…

## Slide 12
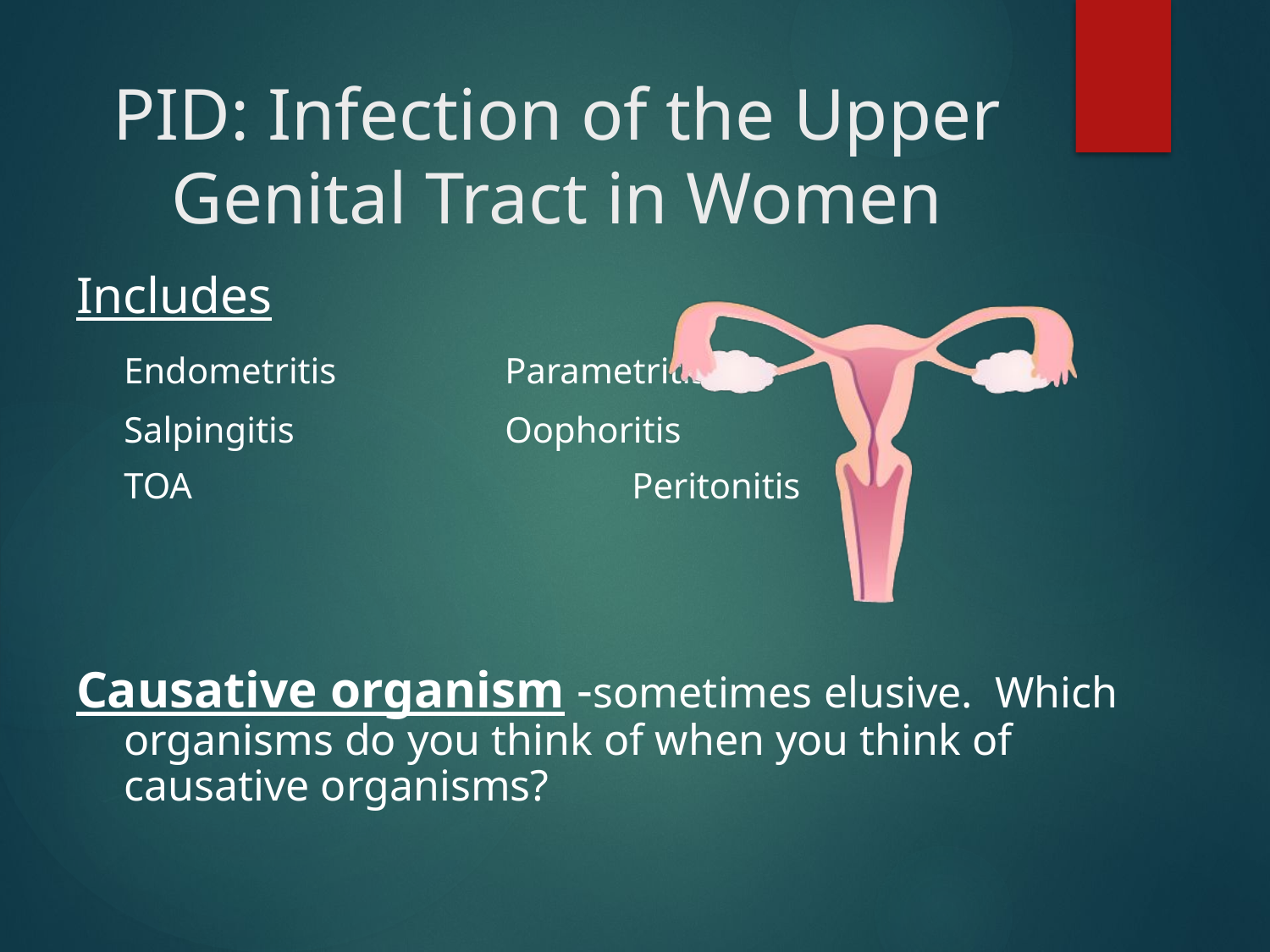

# PID: Infection of the Upper Genital Tract in Women
Includes
	Endometritis		Parametritis
	Salpingitis		Oophoritis
	TOA				Peritonitis
Causative organism -sometimes elusive. Which organisms do you think of when you think of causative organisms?

## Slide 13
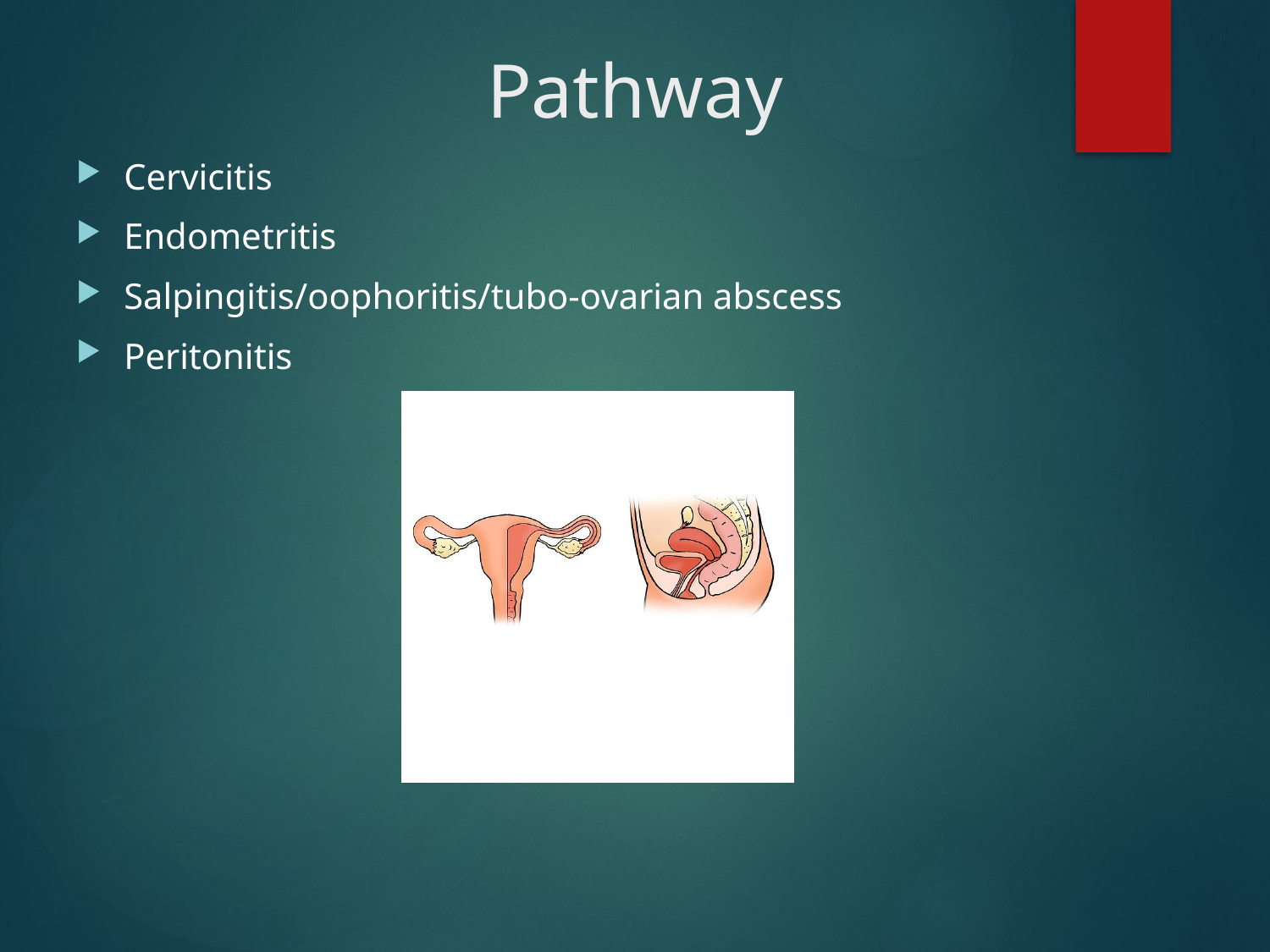

# Pathway
Cervicitis
Endometritis
Salpingitis/oophoritis/tubo-ovarian abscess
Peritonitis

## Slide 14
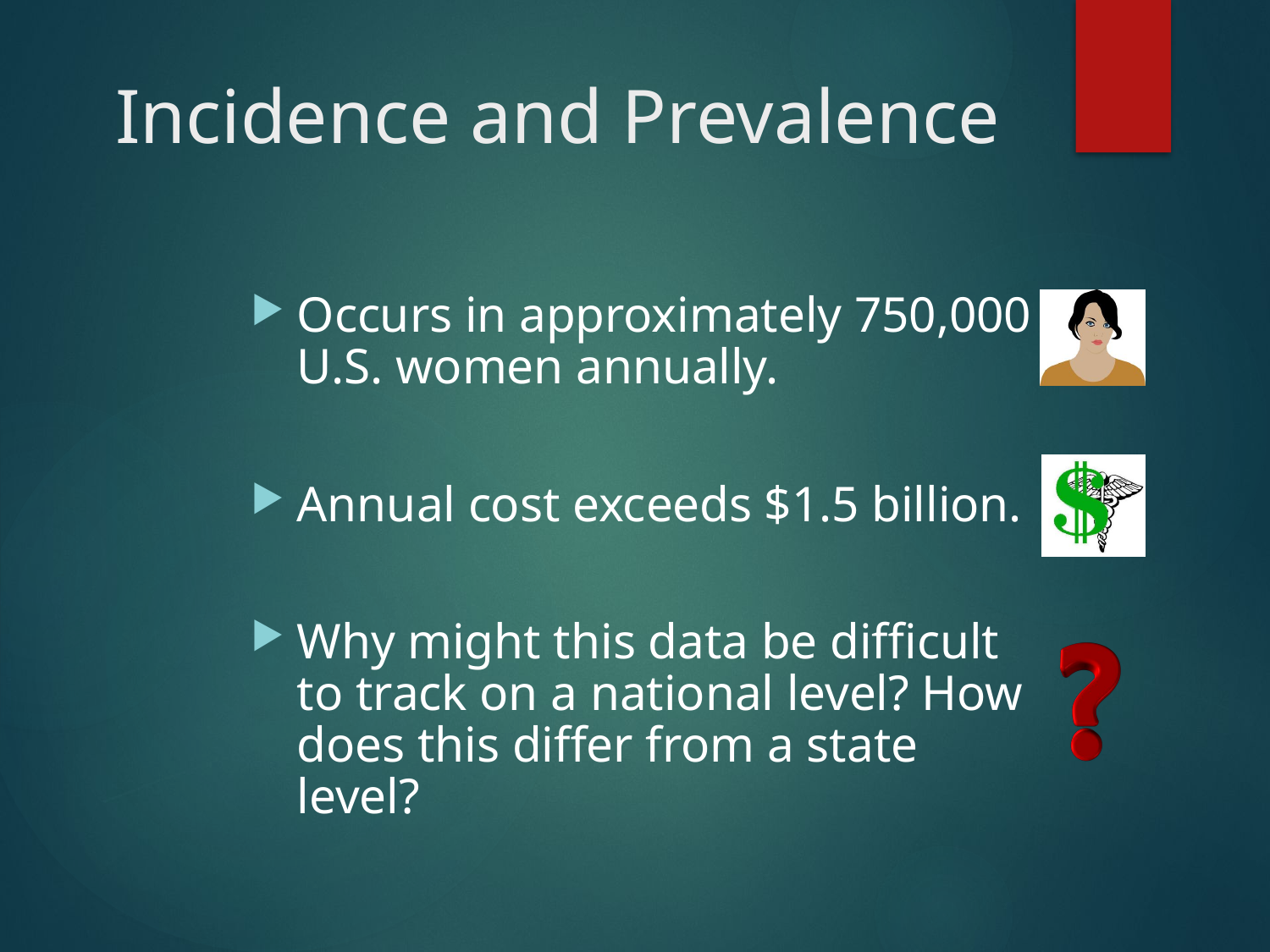

# Incidence and Prevalence
Occurs in approximately 750,000 U.S. women annually.
Annual cost exceeds $1.5 billion.
Why might this data be difficult to track on a national level? How does this differ from a state level?

## Slide 15
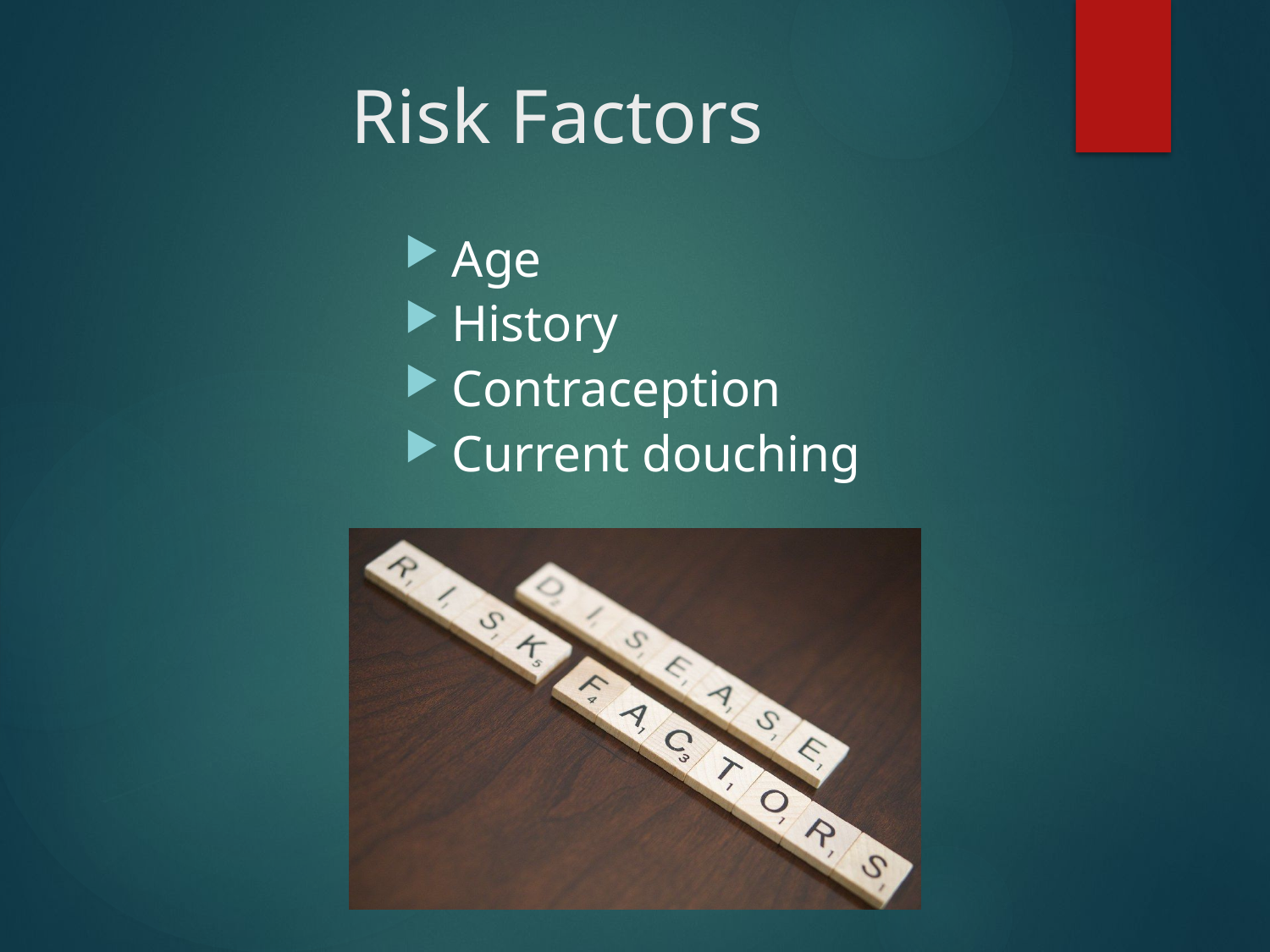

# Risk Factors
Age
History
Contraception
Current douching

## Slide 16
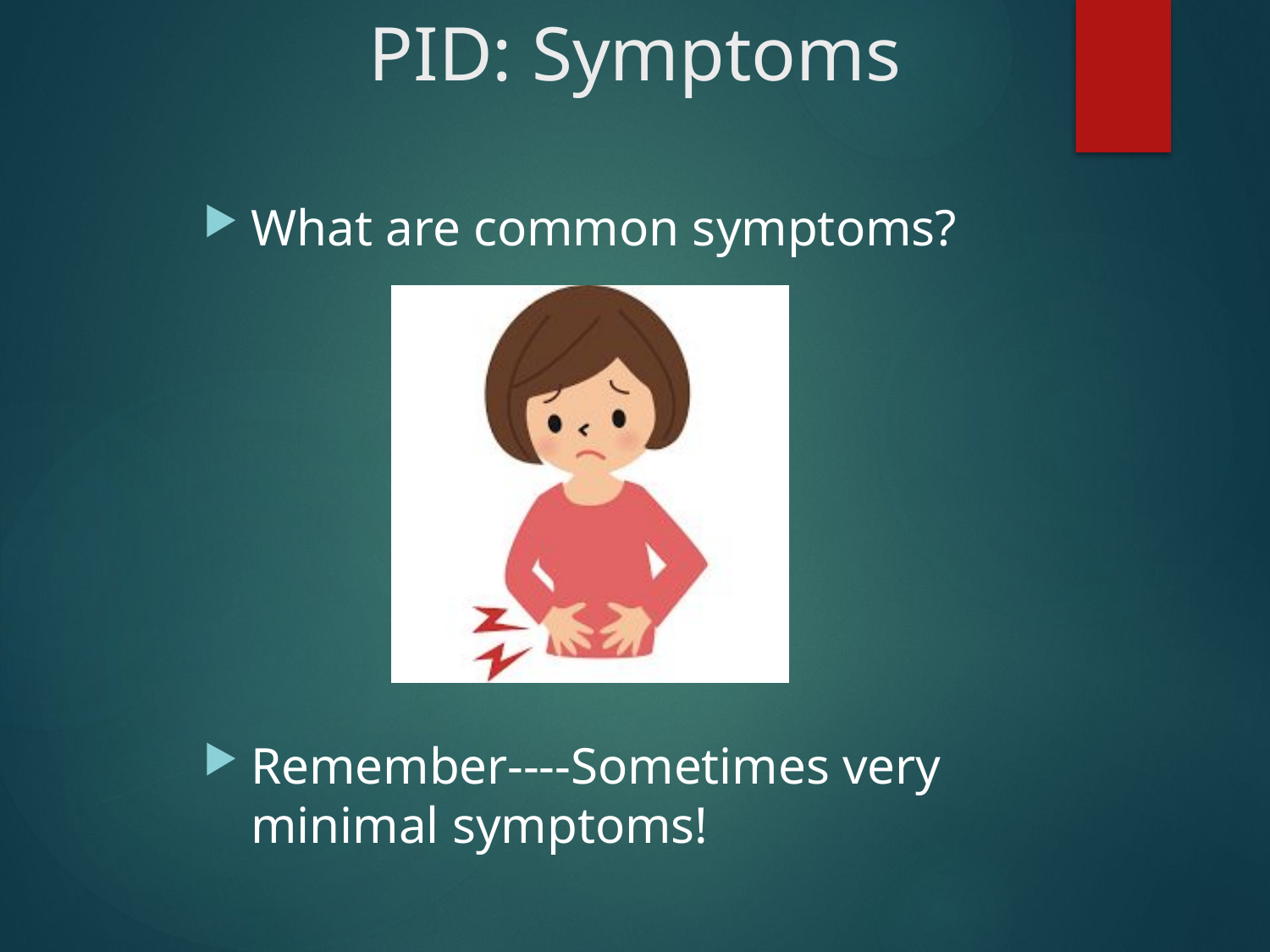

# PID: Symptoms
What are common symptoms?
Remember----Sometimes very minimal symptoms!

## Slide 17
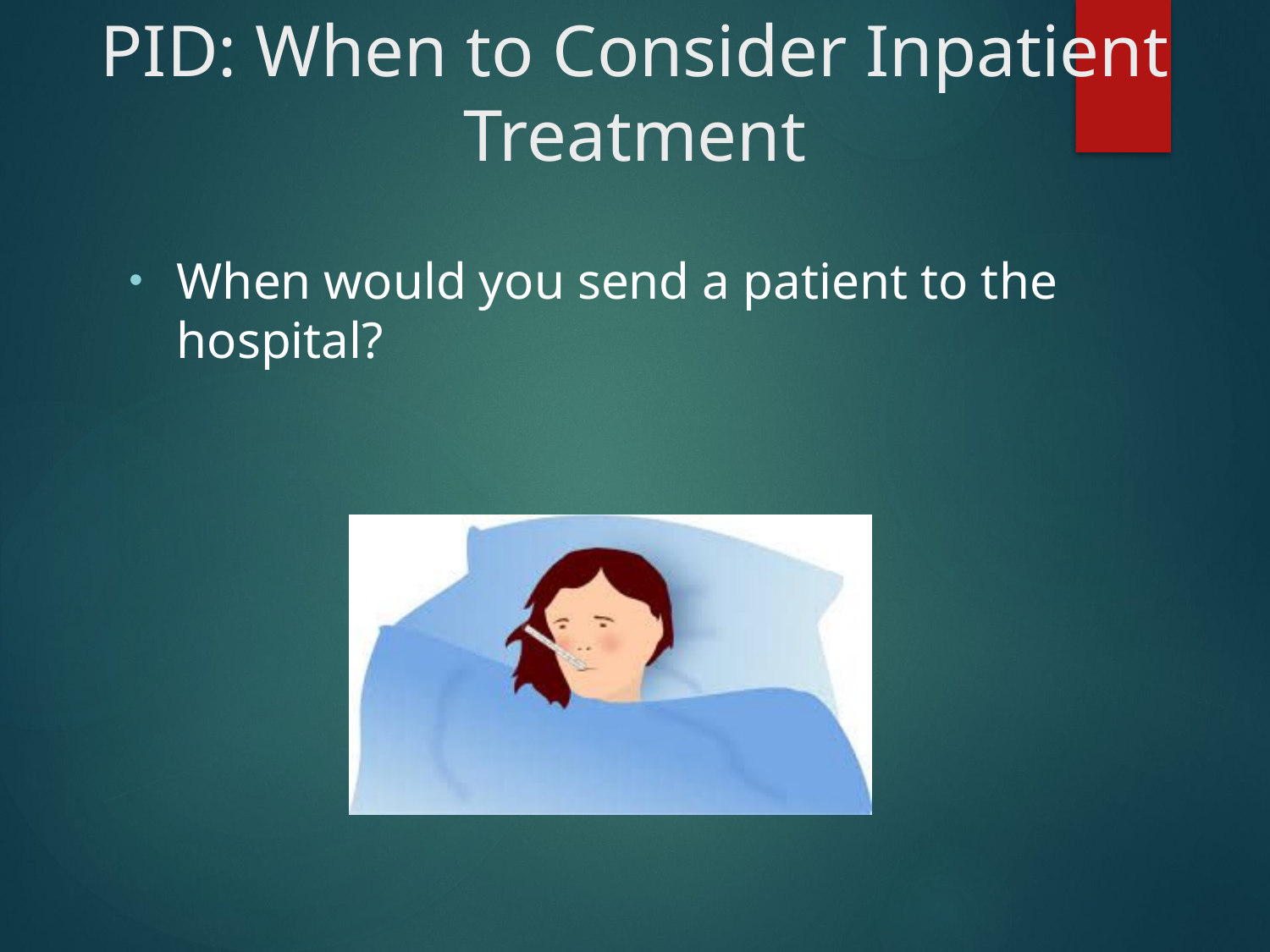

# PID: When to Consider Inpatient Treatment
When would you send a patient to the hospital?

## Slide 18
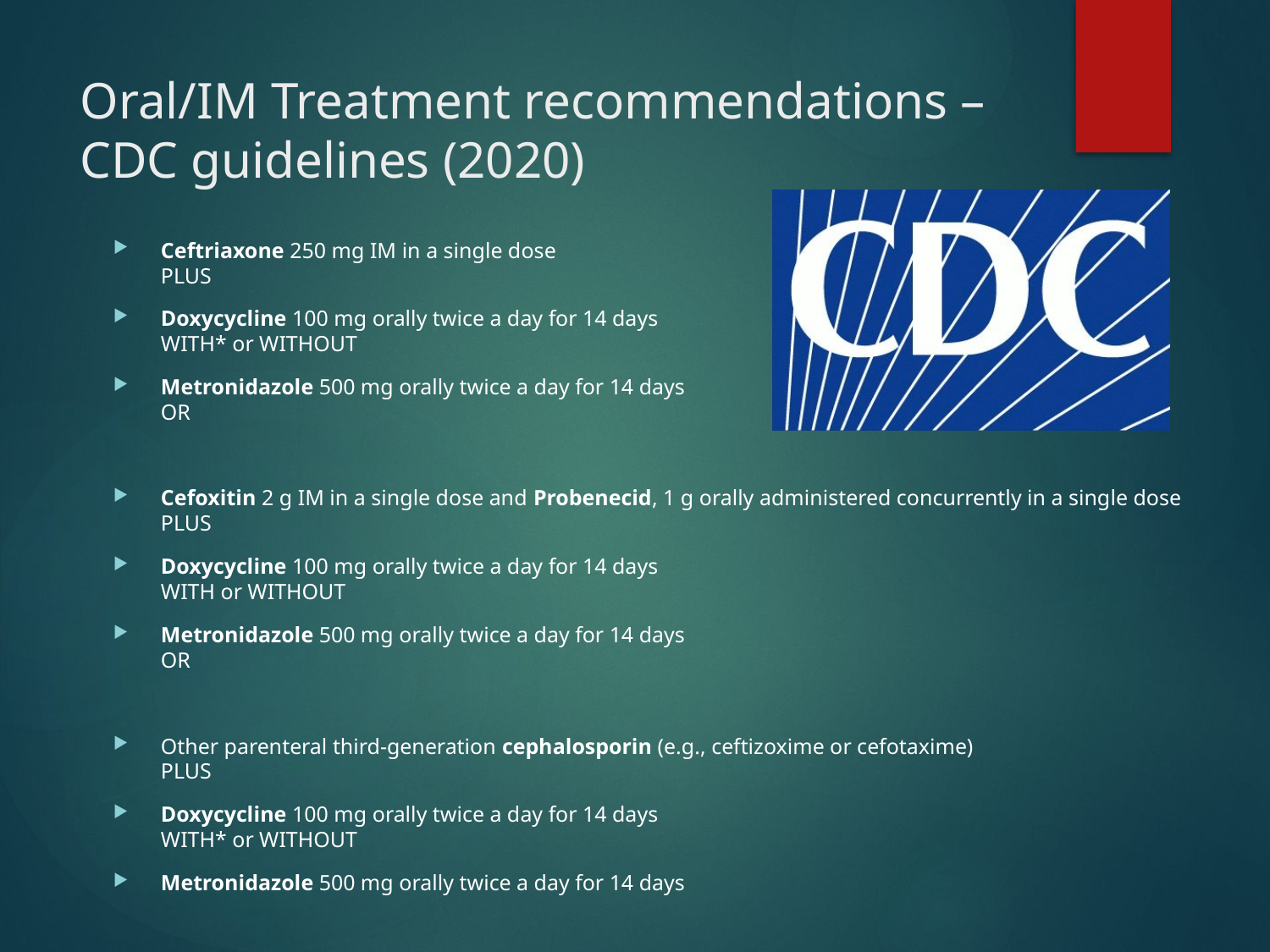

# Oral/IM Treatment recommendations – CDC guidelines (2020)
Ceftriaxone 250 mg IM in a single dose
PLUS
Doxycycline 100 mg orally twice a day for 14 days
WITH* or WITHOUT
Metronidazole 500 mg orally twice a day for 14 days
OR
Cefoxitin 2 g IM in a single dose and Probenecid, 1 g orally administered concurrently in a single dose
PLUS
Doxycycline 100 mg orally twice a day for 14 days
WITH or WITHOUT
Metronidazole 500 mg orally twice a day for 14 days
OR
Other parenteral third-generation cephalosporin (e.g., ceftizoxime or cefotaxime)
PLUS
Doxycycline 100 mg orally twice a day for 14 days
WITH* or WITHOUT
Metronidazole 500 mg orally twice a day for 14 days

## Slide 19
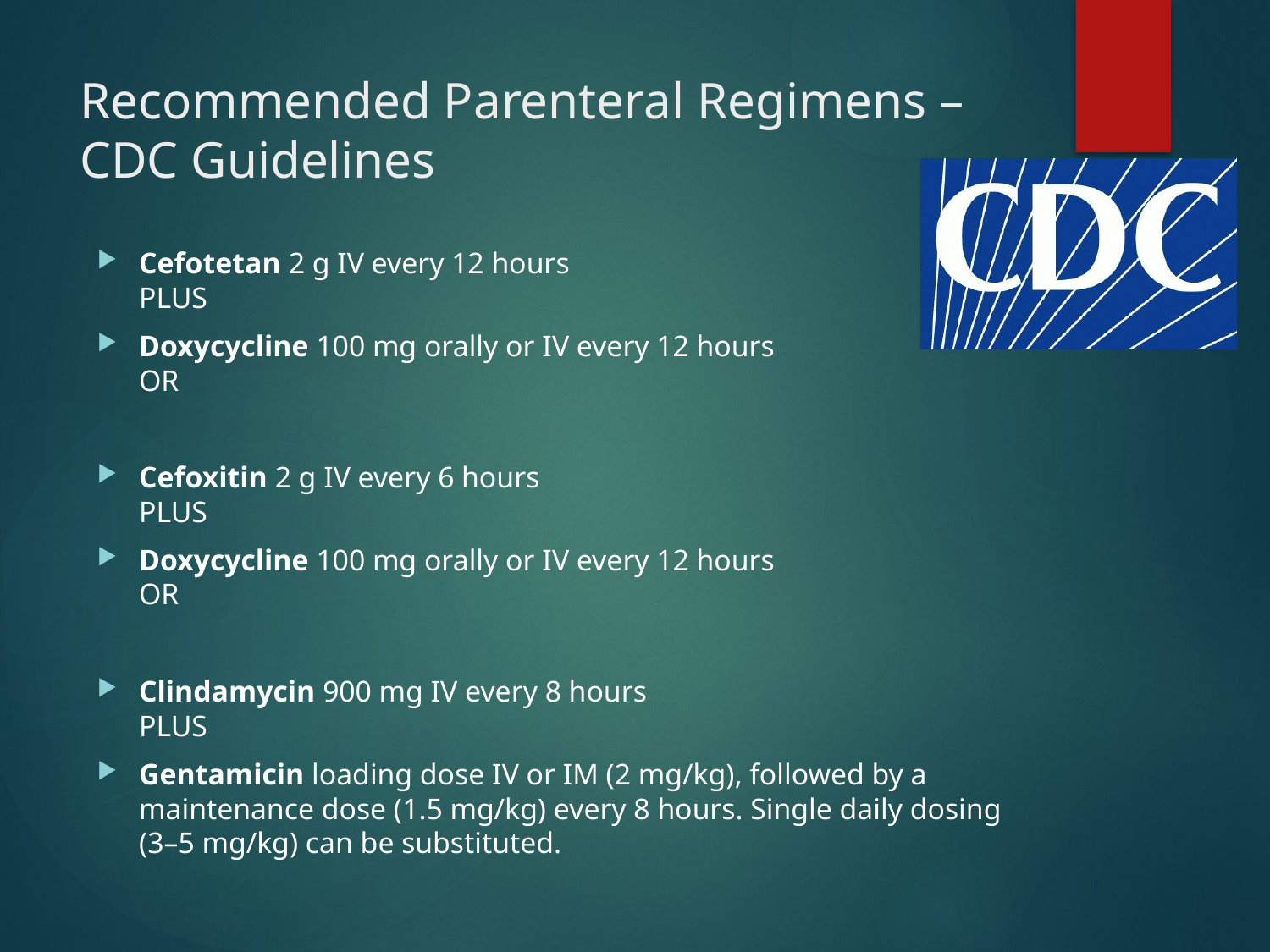

# Recommended Parenteral Regimens – CDC Guidelines
Cefotetan 2 g IV every 12 hours
PLUS
Doxycycline 100 mg orally or IV every 12 hours
OR
Cefoxitin 2 g IV every 6 hours
PLUS
Doxycycline 100 mg orally or IV every 12 hours
OR
Clindamycin 900 mg IV every 8 hours
PLUS
Gentamicin loading dose IV or IM (2 mg/kg), followed by a maintenance dose (1.5 mg/kg) every 8 hours. Single daily dosing (3–5 mg/kg) can be substituted.

## Slide 20
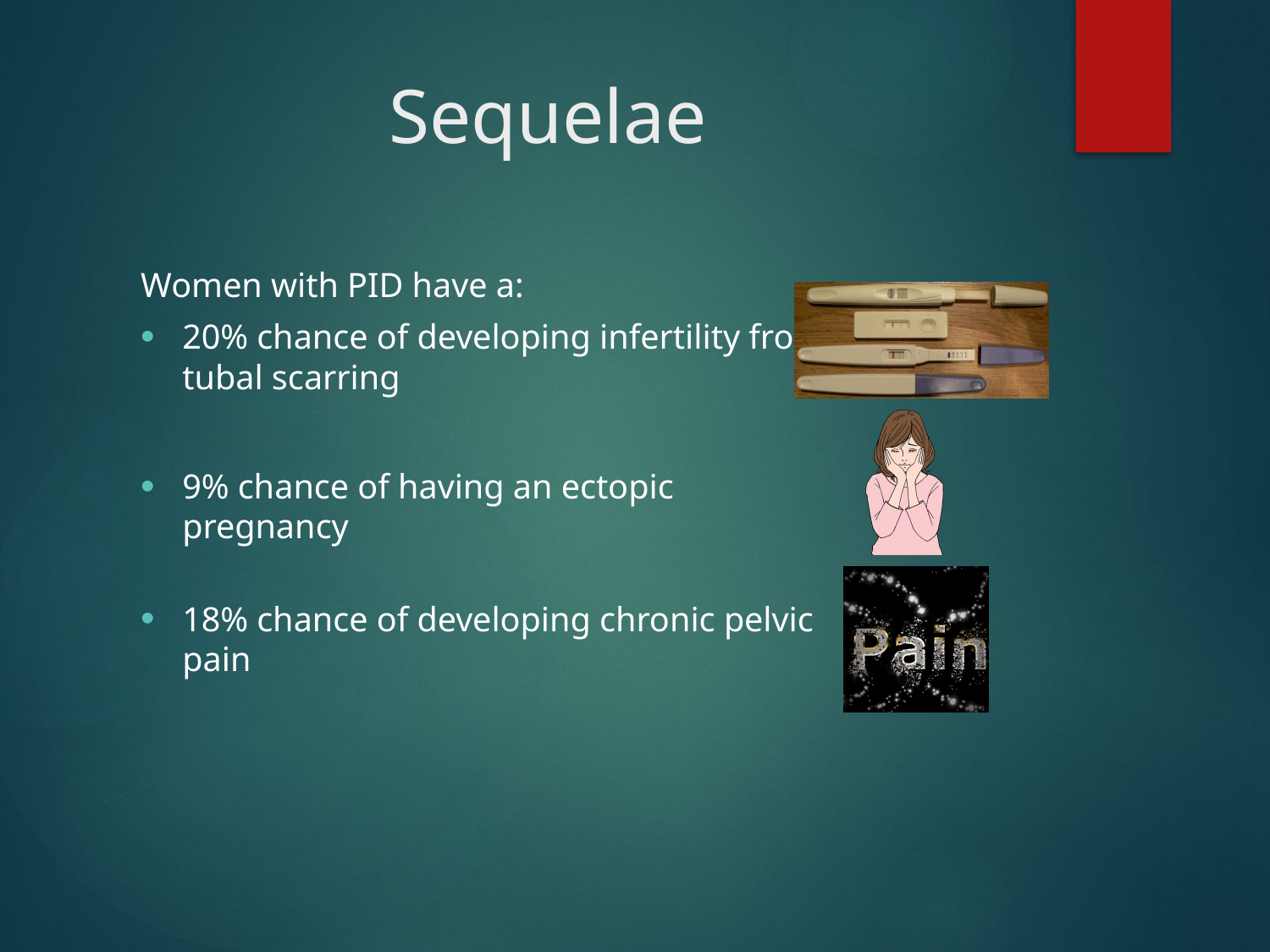

# Sequelae
Women with PID have a:
20% chance of developing infertility from tubal scarring
9% chance of having an ectopic pregnancy
18% chance of developing chronic pelvic pain

## Slide 21
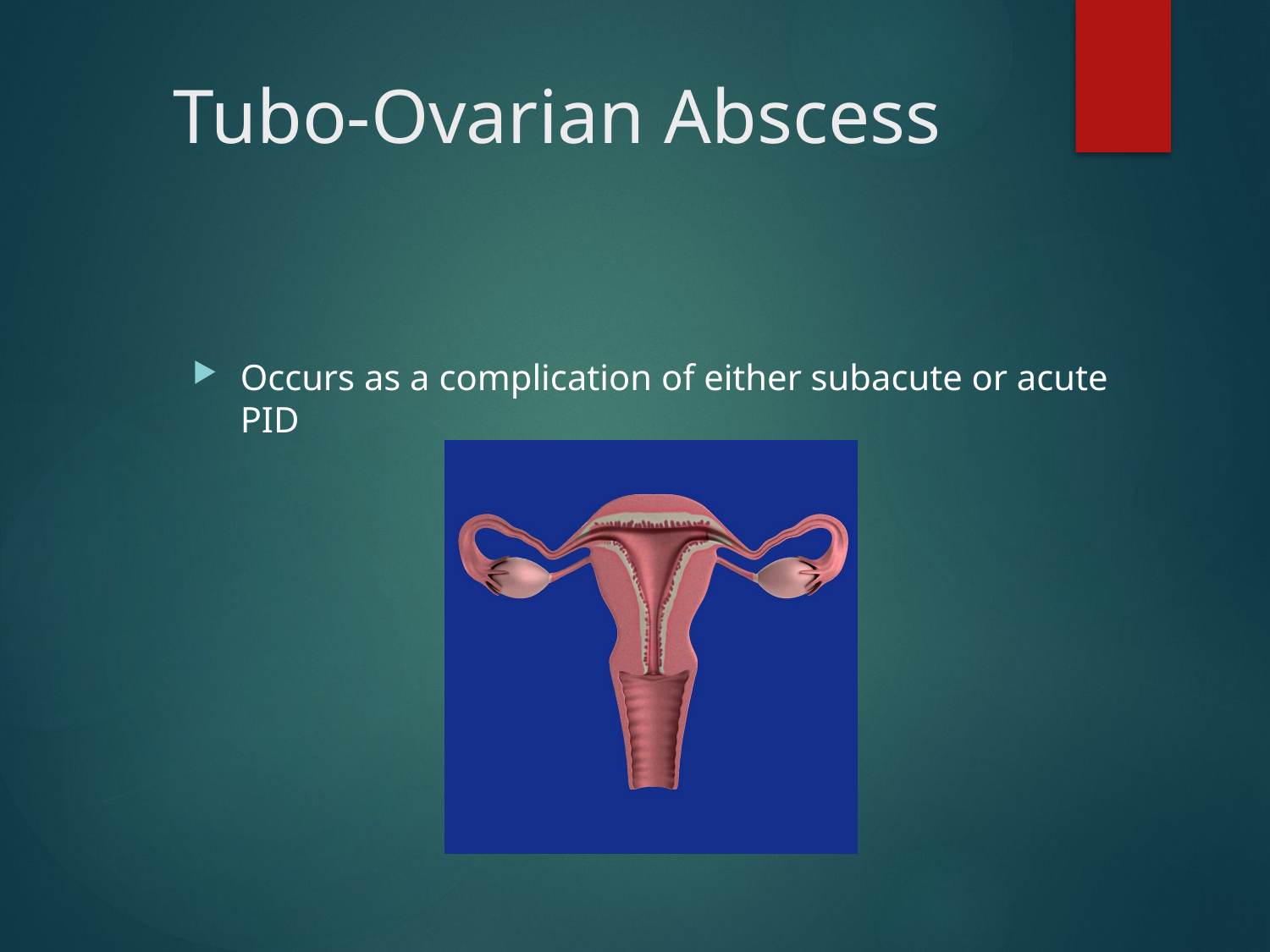

# Tubo-Ovarian Abscess
Occurs as a complication of either subacute or acute PID

## Slide 22
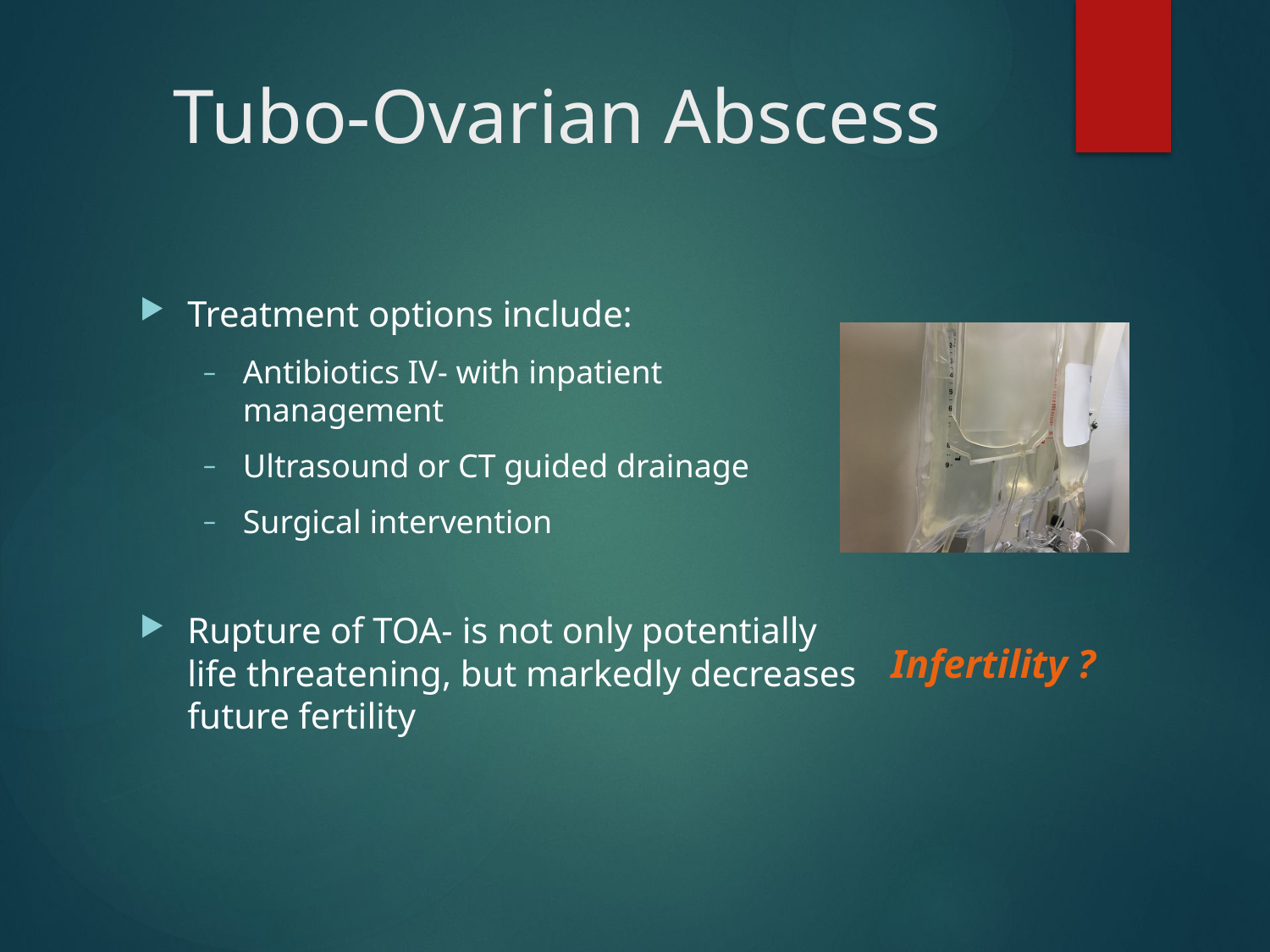

# Tubo-Ovarian Abscess
Treatment options include:
Antibiotics IV- with inpatient management
Ultrasound or CT guided drainage
Surgical intervention
Rupture of TOA- is not only potentially life threatening, but markedly decreases future fertility
Infertility ?

## Slide 23
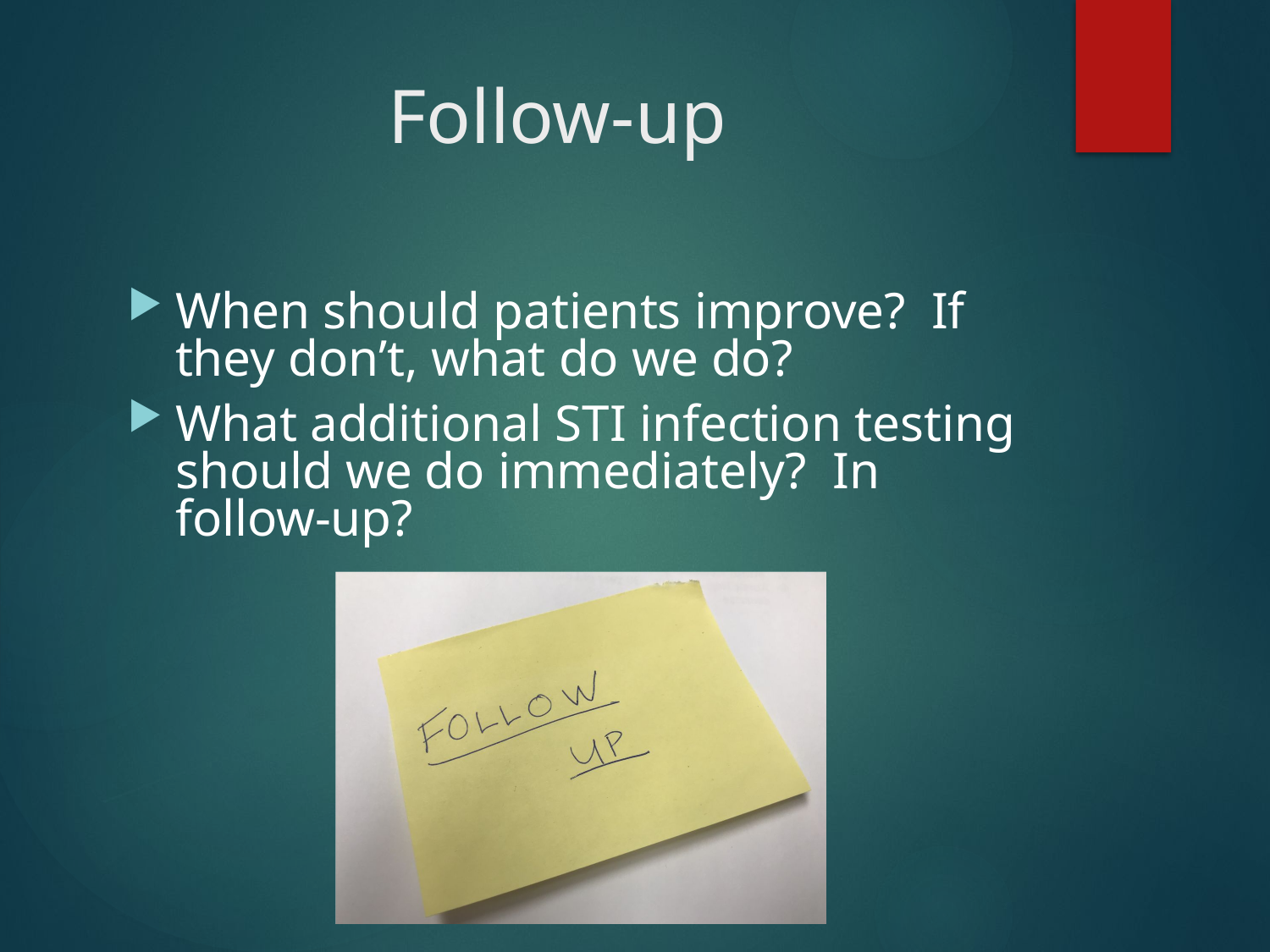

# Follow-up
When should patients improve? If they don’t, what do we do?
What additional STI infection testing should we do immediately? In follow-up?

## Slide 24
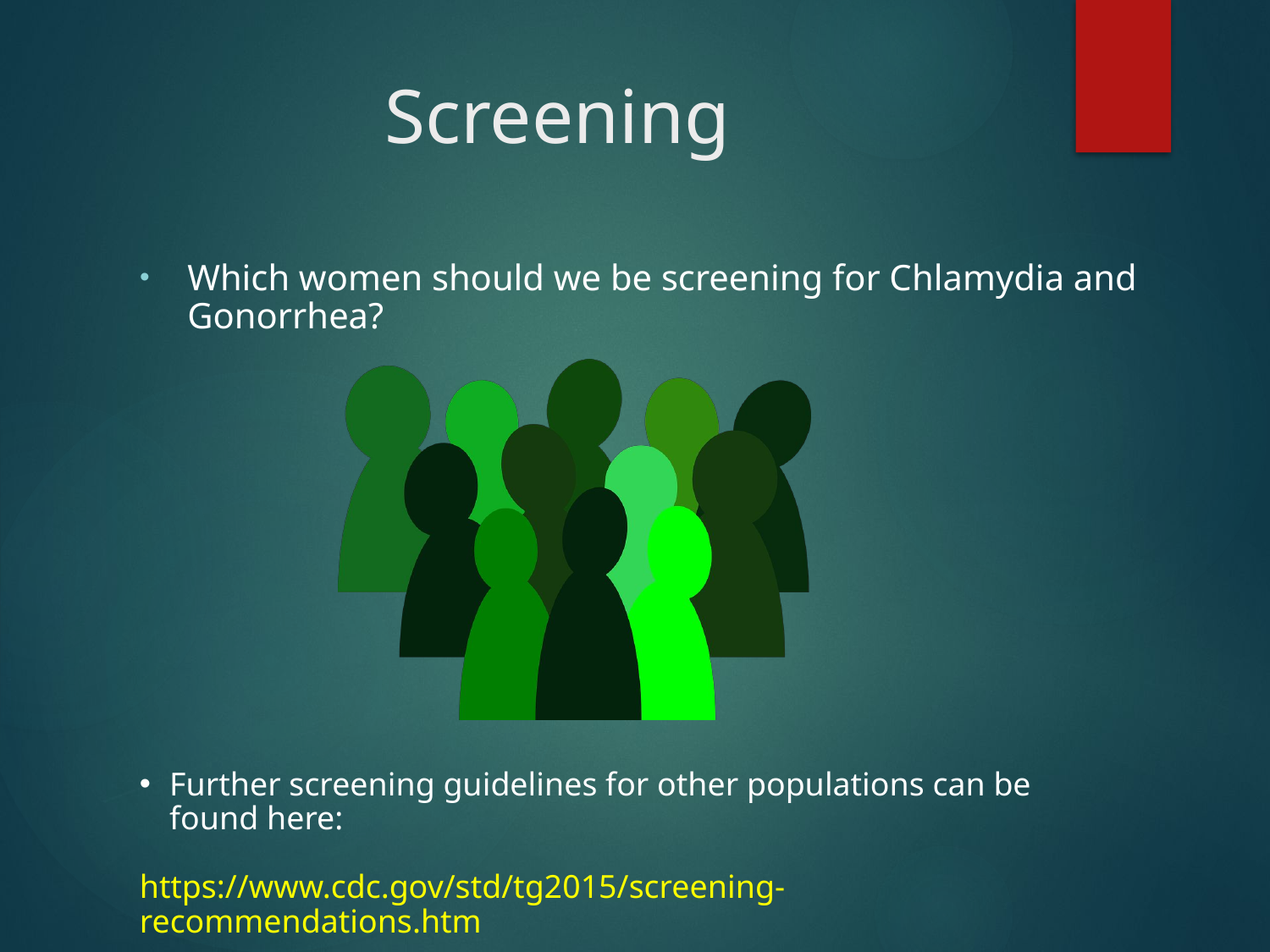

# Screening
Which women should we be screening for Chlamydia and Gonorrhea?
Further screening guidelines for other populations can be found here:
https://www.cdc.gov/std/tg2015/screening-recommendations.htm

## Slide 25
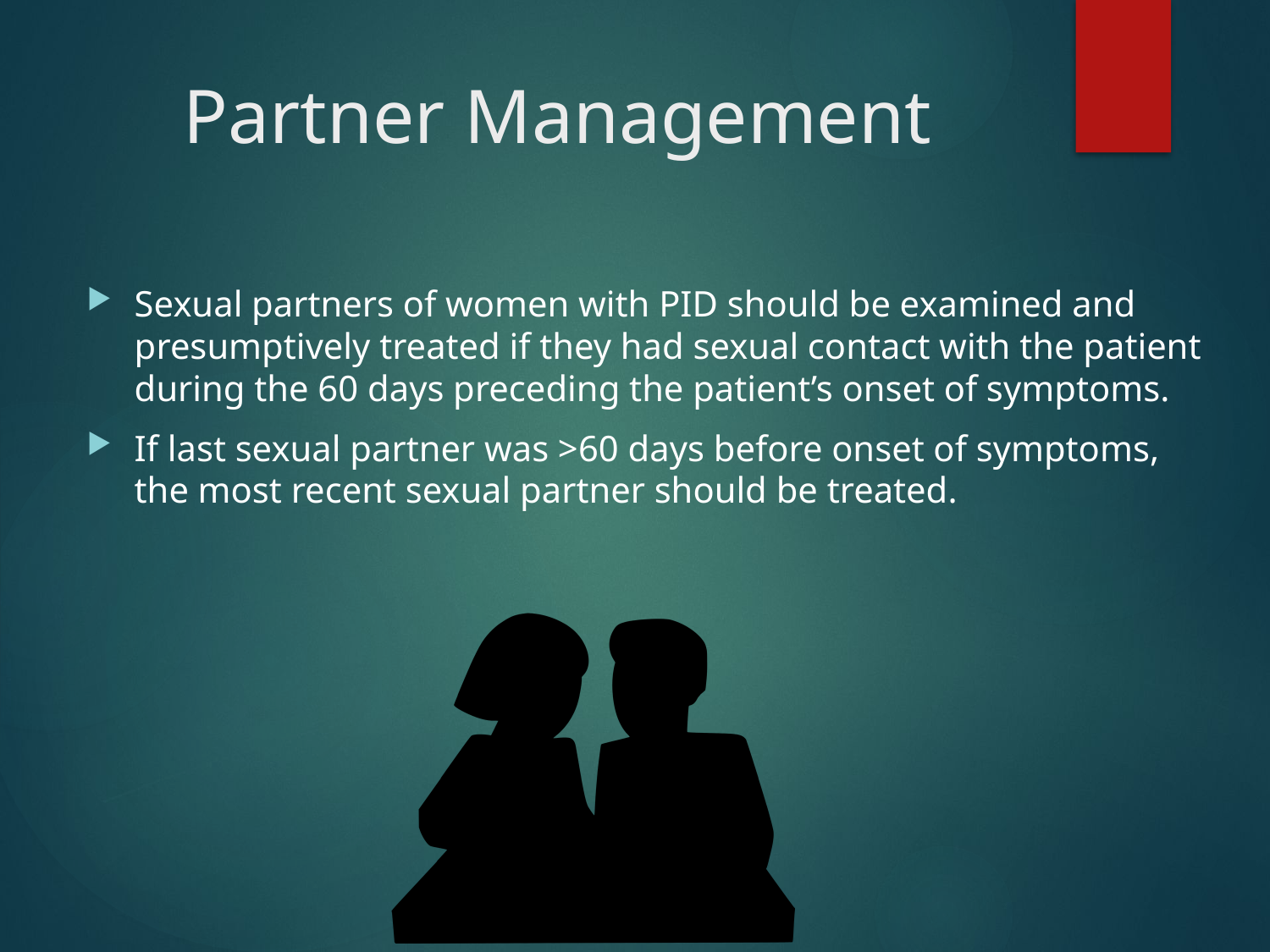

# Partner Management
Sexual partners of women with PID should be examined and presumptively treated if they had sexual contact with the patient during the 60 days preceding the patient’s onset of symptoms.
If last sexual partner was >60 days before onset of symptoms, the most recent sexual partner should be treated.

## Slide 26
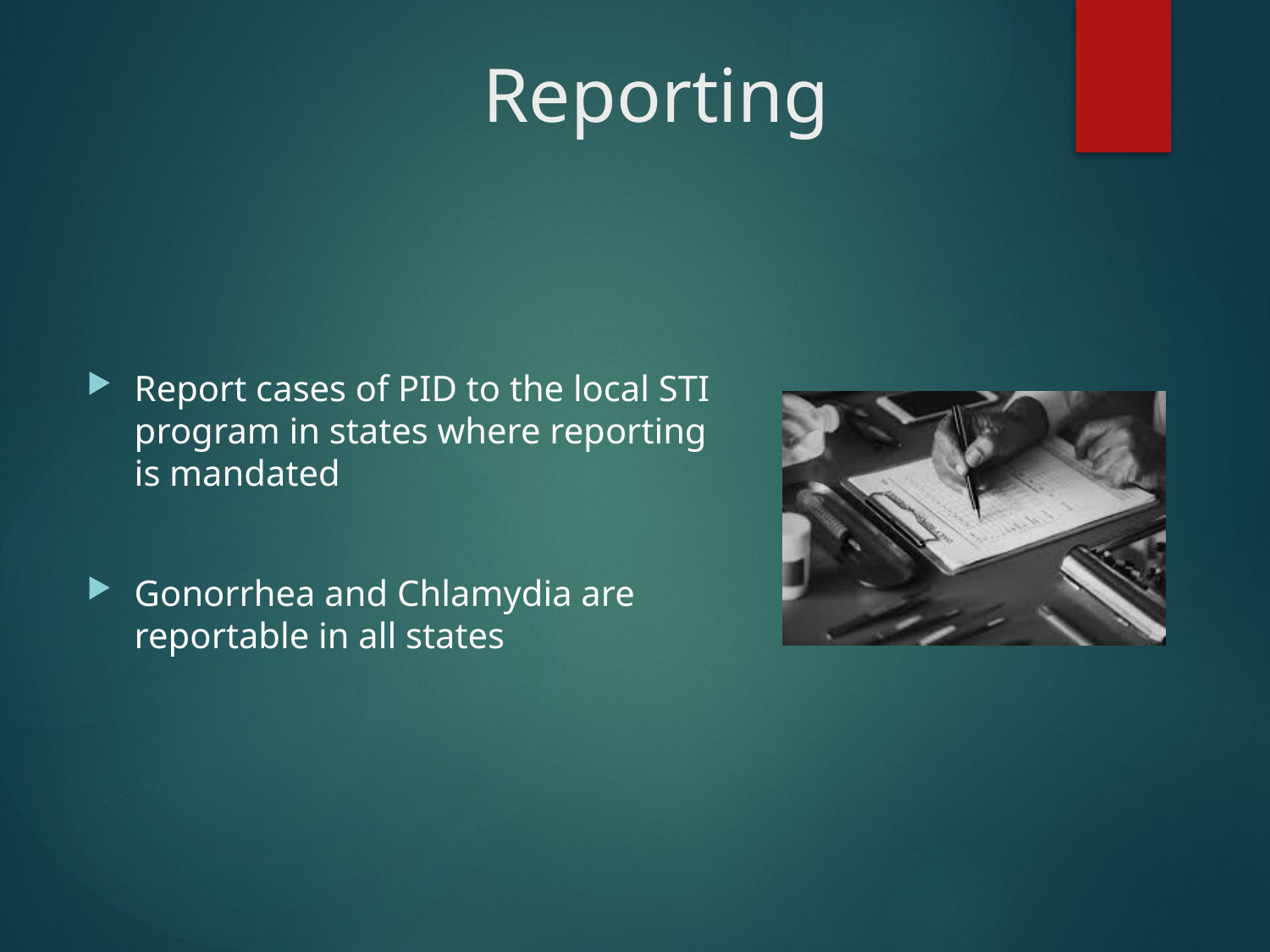

# Reporting
Report cases of PID to the local STI program in states where reporting is mandated
Gonorrhea and Chlamydia are reportable in all states

## Slide 27
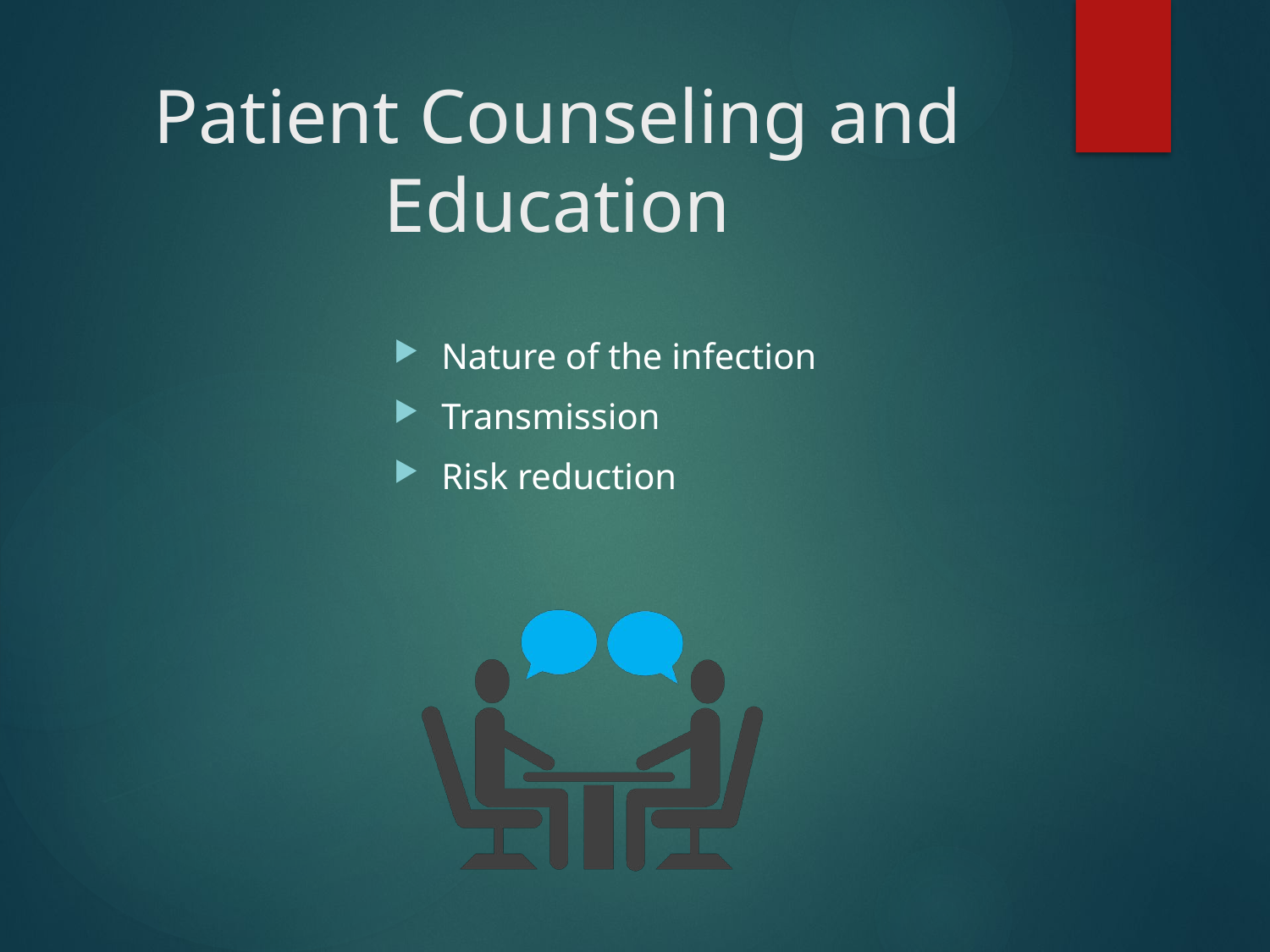

# Patient Counseling and Education
Nature of the infection
Transmission
Risk reduction

## Slide 28
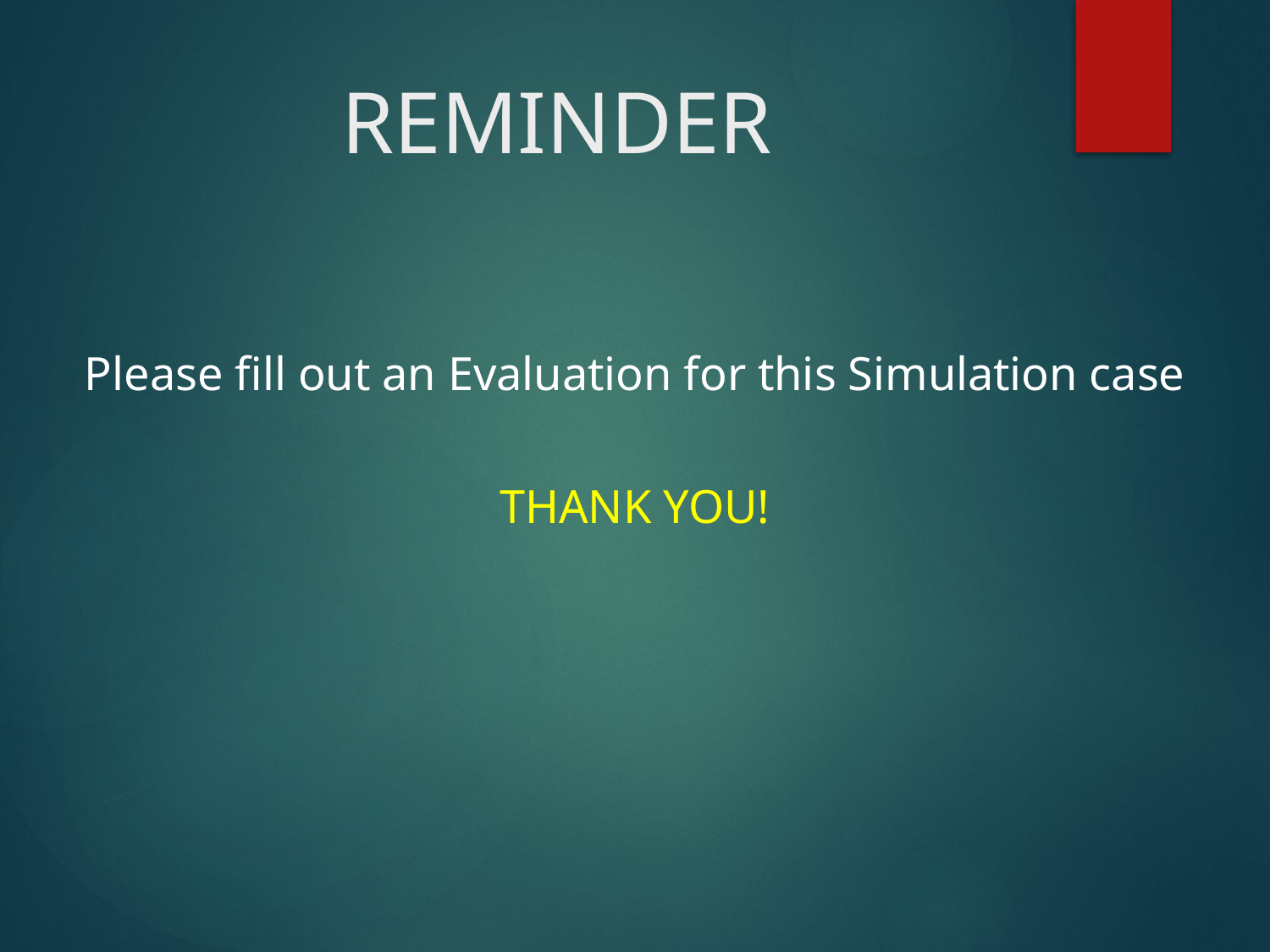

# REMINDER
Please fill out an Evaluation for this Simulation case
THANK YOU!

## Slide 29
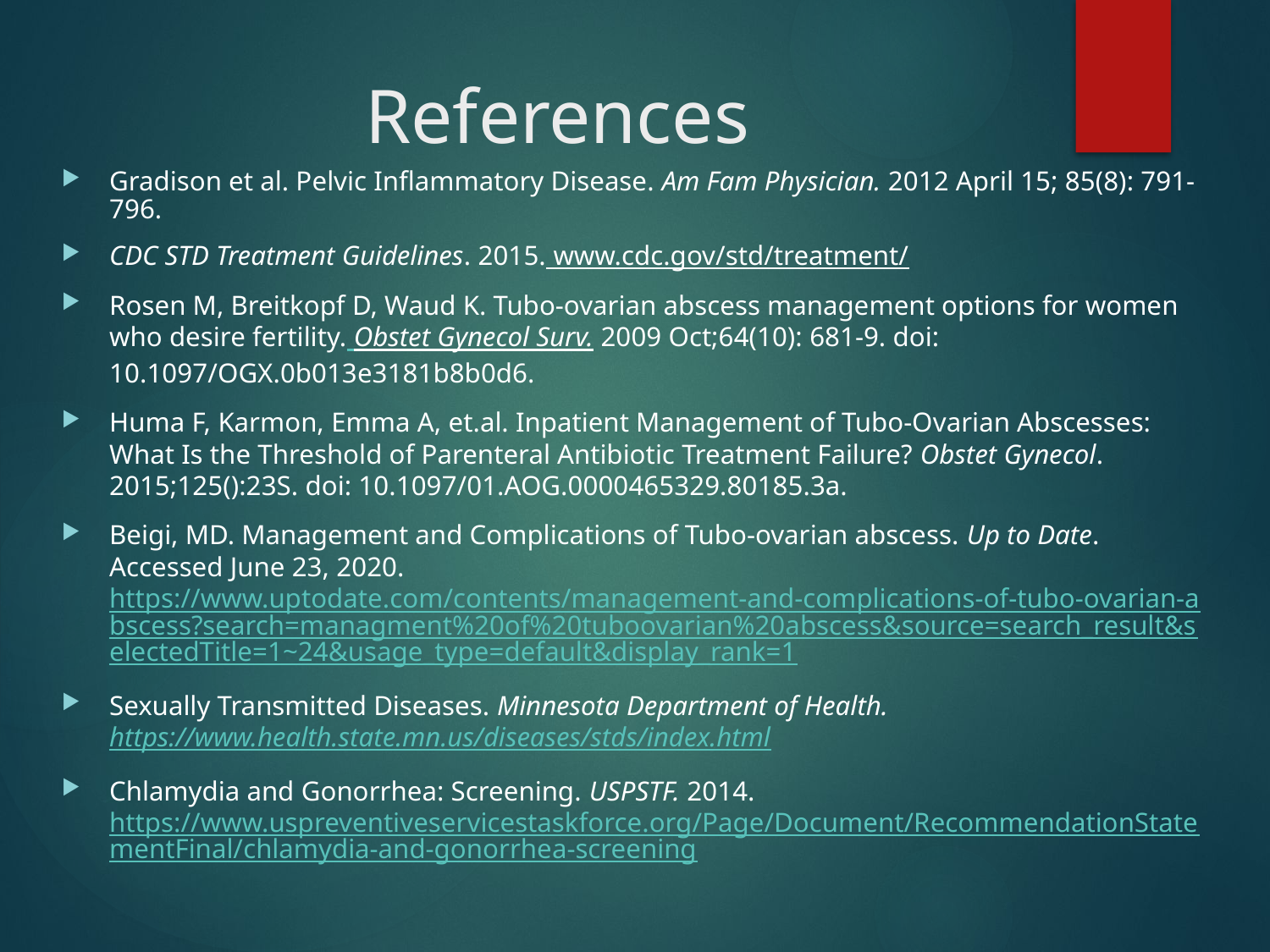

# References
Gradison et al. Pelvic Inflammatory Disease. Am Fam Physician. 2012 April 15; 85(8): 791-796.
CDC STD Treatment Guidelines. 2015. www.cdc.gov/std/treatment/
Rosen M, Breitkopf D, Waud K. Tubo-ovarian abscess management options for women who desire fertility. Obstet Gynecol Surv. 2009 Oct;64(10): 681-9. doi: 10.1097/OGX.0b013e3181b8b0d6.
Huma F, Karmon, Emma A, et.al. Inpatient Management of Tubo-Ovarian Abscesses: What Is the Threshold of Parenteral Antibiotic Treatment Failure? Obstet Gynecol. 2015;125():23S. doi: 10.1097/01.AOG.0000465329.80185.3a.
Beigi, MD. Management and Complications of Tubo-ovarian abscess. Up to Date. Accessed June 23, 2020. https://www.uptodate.com/contents/management-and-complications-of-tubo-ovarian-abscess?search=managment%20of%20tuboovarian%20abscess&source=search_result&selectedTitle=1~24&usage_type=default&display_rank=1
Sexually Transmitted Diseases. Minnesota Department of Health. https://www.health.state.mn.us/diseases/stds/index.html
Chlamydia and Gonorrhea: Screening. USPSTF. 2014. https://www.uspreventiveservicestaskforce.org/Page/Document/RecommendationStatementFinal/chlamydia-and-gonorrhea-screening
