# Supplementary material for: Teaching the Evaluation of Female Pelvic Pain: A Hands-On Simulation to Reinforce Exam Skills and Introduce Transvaginal Ultrasound
Source: MedEdPORTAL. 2021 Jan 25;17:11080. doi: 10.15766/mep_2374-8265.11080 (PMC7830760; doi:10.15766/mep_2374-8265.11080)
Supplement: Supplementary file 1 — Simulation Case.docxDebrief PowerPoint.pptxFaculty Critical Action Checklist.docxStudent Survey.docx [file mep_2374-8265.11080-s001.zip › C. Faculty Critical Action Checklist.docx]

**Faculty Critical Action Checklist**

| Students *(each number correlates to an individual student- record names/notes on performance and/or contributions to the simulation group)* | 1 | 2 | 3 | 4 | 5 | 6 | 7 |
| --- | --- | --- | --- | --- | --- | --- | --- |
| Team dynamics  Perform assigned role  Closed loop communication |  |  |  |  |  |  |  |
| Obtain and interpret vital signs  Request vitals  Monitor vital signs through case |  |  |  |  |  |  |  |
| Obtain focused subjective history  Characteristics of pain  Associated symptoms |  |  |  |  |  |  |  |
| Demonstrate patient centered care  Create rapport  Elicit patient’s perspective  Demonstrate empathy |  |  |  |  |  |  |  |
| Obtain pertinent past medical history  History/risk factors for STI  Menstrual history  Obstetric history  Contraception plan |  |  |  |  |  |  |  |
| Perform appropriate physical exam  HEENT  Cardiac/lungs  Abdomen: include special testing  Pelvic exam  Rectal Exam (discussed) |  |  |  |  |  |  |  |
| Consideration of differential diagnosis |  |  |  |  |  |  |  |
| Request testing  Pregnancy test  CBC with differential  Blood cultures  Vaginal sampling: wet prep, GC/chlamydia  UA  ESR/CRP |  |  |  |  |  |  |  |
| Request imaging  Ultrasound |  |  |  |  |  |  |  |
| Treatment considerations – stabilization  Fluids: 2 large bore IV’s and fluid bolus  Oxygen by nasal canula |  |  |  |  |  |  |  |
| Treatment considerations – management  Antibiotic choice/mode of delivery  Pain management  OB/Gyn consult / surgical drainage |  |  |  |  |  |  |  |
| Follow-up plan of care  Inpatient  Outpatient  Potential sequelae |  |  |  |  |  |  |  |
