# Supplementary material for: Teaching the Evaluation of Female Pelvic Pain: A Hands-On Simulation to Reinforce Exam Skills and Introduce Transvaginal Ultrasound
Source: MedEdPORTAL. 2021 Jan 25;17:11080. doi: 10.15766/mep_2374-8265.11080 (PMC7830760; doi:10.15766/mep_2374-8265.11080)
Supplement: Supplementary file 1 — Simulation Case.docxDebrief PowerPoint.pptxFaculty Critical Action Checklist.docxStudent Survey.docx [file mep_2374-8265.11080-s001.zip › D. Student Survey.docx]

**Student Survey**

**Tubo-Ovarian Abscess Simulation Case**

1. Who was your faculty facilitator?
2. Please rank the following:

|  | Strongly Disagree | Disagree | Neither Agree nor Disagree | Agree | Strongly Agree |
| --- | --- | --- | --- | --- | --- |
| The case history was clear |  |  |  |  |  |
| The simulation was realistic |  |  |  |  |  |
| Participating in this simulation session enhanced my knowledge of the case presented |  |  |  |  |  |
| The debriefing session was valuable |  |  |  |  |  |
| The simulation case was valuable |  |  |  |  |  |

1. Did your simulation have technical problems?
   - Yes
   - No
2. If yes, what technical problems did you experience?
3. Please list one thing you liked about this session and would NOT change for future students.
4. Please list one thing about this simulation that could be improved for future students.
5. Do you have any additional comments?
