# Supplementary material for: Teaching the Evaluation of Female Pelvic Pain: A Hands-On Simulation to Reinforce Exam Skills and Introduce Transvaginal Ultrasound
Source: MedEdPORTAL. 2021 Jan 25;17:11080. doi: 10.15766/mep_2374-8265.11080 (PMC7830760; doi:10.15766/mep_2374-8265.11080)
Supplement: Supplementary file 1 — Simulation Case.docxDebrief PowerPoint.pptxFaculty Critical Action Checklist.docxStudent Survey.docx [file mep_2374-8265.11080-s001.zip › _Educational_Summary_Report_11080.pdf]

MedEdPORTAL® The AAMC Journal of Teaching and Learning Resources

Original Publication

Open Access

Teaching the Evaluation of Female Pelvic Pain: A Hands-On Simulation to Reinforce Exam Skills and Introduce Transvaginal Ultrasound

Jennifer Pearson, MD*, Amy Greminger, MD, Emily Onello, MD, Sandy Stover, MD

*Corresponding author: jpearso1@d.umn.edu

Abstract

Introduction: Reproductive-age female patients commonly seek evaluation for pelvic pain in a variety of health care settings. Thus, teaching medical students how to effectively evaluate female pelvic pain is a necessary part of medical education. There are limited opportunities, however, to reinforce the needed skills for this common but sensitive presentation that can be anxiety-producing for preclinical students. **Methods:** The case involved a 23-year-old female presenting with lower abdominal/pelvic pain. Students performed the necessary history, physical examination, cervical sampling, and transvaginal ultrasound evaluation to make the diagnosis of pelvic inflammatory disease (PID) complicated by a tubo-ovarian abscess. The 30-minute simulated patient encounter was followed by a 30-40 minute faculty-led debrief. **Results:** This simulation case has been sustained in the curriculum since 2011 for 65 students per year. Through use of a faculty critical action checklist, debrief discussion, examination performance, and student evaluation feedback, this simulation has demonstrated effectiveness. Of students, 93 of 193 students (48%) who participated in the simulation case from 2018-2020 completed a survey in which they rated the degree of agreement with statements about the simulation based on a 5-point Likert Scale (1 = *strongly disagree*, 5 = *strongly agree*). All questions had a mean response of 4.5-4.8 in 2018-2020, demonstrating the consistent agreement by students of the clarity, fidelity, and knowledge-enhancing value of the simulation. **Discussion:** This simulation provided a useful opportunity and a safe environment for preclinical medical students to acquire knowledge and skills necessary to evaluate a female patient with pelvic pain and PID.

Keywords
Simulation, Female Pelvic Exam, Acute Abdomen, Transvaginal Ultrasound, Sexually Transmitted Infection, Emergency Medicine, Family Medicine, OB/GYN, Women's Health

Educational Objectives

By the end of this activity, learners will be able to:

1. Demonstrate the ability to elicit a patient history given the presentation of female pelvic pain.
2. Demonstrate empathy for a patient suffering from a painful condition such as pelvic inflammatory disease (PID).
3. Assess a female patient with a targeted abdominal and pelvic exam through performance or observation.
4. Develop a differential diagnosis for the presenting history and physical findings.
5. Discuss the utility of transvaginal ultrasound in the evaluation of female pelvic pain.
6. Demonstrate knowledge of pathophysiology, risk factors, clinical presentation, testing, diagnosis, management, reporting, and complications of PID and tubo-ovarian abscess.

Citation:
Pearson J, Greminger A, Onello E, Stover S. Teaching the evaluation of female pelvic pain: a hands-on simulation to reinforce exam skills and introduce transvaginal ultrasound. *MedEdPORTAL*. 2021;17:11080. https://doi.org/10.15766/mep_2374-8265.11080

Introduction

The presentation of acute pelvic pain in reproductive-aged women is common in primary care, urgent care, and emergency department settings.[1,2] Numerous conditions can cause acute pelvic pain in young women, with a broad differential diagnosis that includes ovarian torsion, ectopic pregnancy, urinary tract infection, acute appendicitis, distal ureteral stone, and pelvic inflammatory disease (PID) among others. PID should be suspected in any young female who presents with pelvic pain, and other concerning diagnosis requiring urgent/emergent attention also need to be ruled out.

Copyright © 2021 Pearson et al. This is an open-access publication distributed under the terms of the Creative Commons Attribution license.

In 2001, there were more than 750,000 cases of PID in the United States. Over the past 10 years, the rates have decreased, but PID is still frequently encountered in clinical practice.[1,2] Because the diagnosis of PID is made clinically, the managing physician must have sufficient knowledge and skill to obtain the essential elements of the patient's history and physical examination while using imaging and laboratory testing to further confirm the diagnosis and rule out other possible pathology.[3] Due to PID's infectious etiology, physicians must be able to competently perform techniques of cervical sampling in order to appropriately test for potential causative pathogens. This clinical acumen in evaluating PID is especially important because of the potential short-term complications of PID, which include tubo-ovarian abscess or pelvic abscess. In addition, PID has the potential to result in significant long-term complications such as chronic pelvic pain, impaired fertility, and an increased future risk of ectopic pregnancy.

Thus, teaching medical students how to adequately evaluate and examine female patients with pelvic pain is an important part of undergraduate medical education. Teaching basic physical exam skills is foundational in medical education; however, there are limited opportunities in many undergraduate medical programs to reinforce these basic examination skills prior to the beginning of clerkship experiences.[4-8] Because of this, student anxiety can result when needing to perform these exams on real patients, with limited prior practice, and in sensitive and difficult clinical situations.[9,10] In addition, familiarizing preclinical medical students with modalities with which to evaluate pelvic pain initiates necessary clinical thinking on how to approach and work up this common patient presentation.[11]

This simulation-based learning module contributed to existing medical education literature by offering a novel combination of learning activities and learning objectives for preclinical medical students evaluating acute pelvic pain in a young female patient. A number of excellent medical education resources exist that teach basic pelvic exam skills.[2-9] An additional *MedEdPORTAL* publication provided useful educational material on the topic of PID using a problem-based learning case.[12] Another publication, intended for use by OB/GYN residents, included a simulation case of a 17-year-old female patient with a first trimester septic abortion.[13] The authors are unaware, however, of any publication to date that targets preclinical medical students and integrates the multiple elements of history-taking, female pelvic examination skills with swab/sample collection, and point-of-care pelvic ultrasound for the goal of teaching the diagnosis and management of PID. The simulation case presented here has been time-tested with a decade of durability in the home institution's undergraduate medical curriculum. It offered a novel simulation case that reinforced the fundamental clinical competencies that are necessary to diagnose the common gynecological condition of PID along with one of its potential serious complications, the tubo-ovarian abscess.

Methods

Development

We developed this simulation case for second-year medical students to complete just prior to beginning clinical clerkships. At the University of Minnesota Medical School Duluth campus, this case was run during the Hormone and Reproductive Medicine Course in the spring of the second year and was coordinated with the underlying pathophysiology being taught in the course.

This case was first piloted in April of 2011 and has been run annually since. All students who participated in this simulation had received prior instruction on the following: (1) taking a patient history and beginning differential diagnosis formation, (2) performing female abdominal and pelvic exams, (3) a lecture introducing ultrasound basics, (4) hands-on practice with ultrasound techniques in other regions of the body (no prior exposure to transvaginal probe), and (5) experience with simulation cases within each core course throughout first and second years of medical school.

Besides the above-mentioned prior instruction in history-taking, differential formation, and examination skills, no specific prework was required by students for this simulation. Curricular components within the Hormone and Reproductive Medicine Course reiterated the underlying anatomy, physiology, microbiology, and pathology of the female reproductive tract. Preparation required by each clinical faculty for this case involved reviewing the case (Appendix A) and the debrief PowerPoint (Appendix B). Faculty facilitators for this case also performed a transvaginal ultrasound on the accompanying pelvic model prior to the simulation case onset so as to familiarize themselves with the equipment and the pathologic findings.

The case involved a 23-year-old female presenting with lower right-side abdominal/pelvic pain. Students performed the necessary history, physical examination, cervical sampling, and transvaginal ultrasound evaluation to make the diagnosis of PID complicated by a tubo-ovarian abscess.

Equipment

This case required the following equipment:

- Simulation mannequin (with clothing and wig to simulate a female age 23)

Copyright © 2021 Pearson et al. This is an open-access publication distributed under the terms of the Creative Commons Attribution license.

- Blood pressure cuff and sphygmomanometer
- Cardiac monitor leads
- SpO2 monitor
- Nasal oxygen cannula
- IV Pole and IV
- Crash cart including a medicine drawer
- Pelvic trainer model (on which students can perform a female pelvic exam)
- Vaginal ring contraceptive device
- Small speculum
- Gonorrhea/chlamydia testing materials (swab)
- Wet prep testing materials (swab)
- Female ultrasound training pelvis with correlating pathology
- Bedside ultrasound with transvaginal probe (with ultrasound gel and condom covering)
- Hand sanitizer
- Gloves, all sizes
- Faculty checklists (Appendix C) on clipboards for each faculty, one for each small group

Personnel
This simulation case required the following personnel:

- Simulation technician: ran technical components of the case, responded to students' questions/actions.
- Simulation center staff: gave prebrief to students on safety, scenario, and specific equipment available for this activity (this person could be the same as the simulation technician).
- Medical school faculty: observed students and evaluated student performance with the faculty critical action checklist (Appendix C), stepped into the simulation to help guide students through pelvic examination and transvaginal ultrasound, and facilitated the simulation debrief with the entire learning community of students.
- Second-year medical student learners.

Implementation
Approximately 65 medical students in each second-year class were subdivided into five learning communities of 12-13 students per group. For this simulation, each learning community was then split in half, leaving six to seven students to perform the simulation case as a team. Thirty minutes was allocated for each team, with a total of 1 hour allotted to run the entire learning community through the simulation. One clinical faculty member acted as a facilitator for each learning community.

In this simulation experience, each team entered the simulation center and received a short standardized prebrief on student roles, psychological safety, and the available equipment for the scenario. The available simulation mannequin did not allow for pelvic exams, but a pelvic exam task trainer was positioned near the mannequin and students were informed that the task trainer was available if a pelvic exam was needed to complete the simulation. Equipment needed to perform a speculum pelvic examination and cervical sampling for sexually transmitted infection (STI) testing was placed next to the mannequin and covered by a sheet. An ultrasound task trainer and bedside ultrasound machine—not specifically mentioned to students as part of the available equipment so as not to give away needed modality of evaluation for the patient—were also available in this simulation; they were covered by a sheet and positioned away from the immediate area of the scenario.

Students entered the simulation by positioning themselves around the simulation mannequin, working through the case as outlined on the simulation case template (Appendix A). The facilitating faculty remained behind a one-way glass during the initial patient assessment of the simulation, assessing the small group by utilization of the faculty critical action checklist (Appendix C).

When the students determined the need for a pelvic exam, the attending faculty present stepped into the simulation area to offer guidance, reinforcing the correct way to drape and perform a female pelvic exam. The attending faculty also guided students through the cervical sampling options and techniques as the students simulated this sampling on the pelvic model. After the pelvic exam and sampling, the faculty facilitator stepped away from the simulation to allow student discussion about differential diagnosis and management of the patient.

Once students voiced the need for further patient evaluation (most, but not all groups identified ultrasound as the desired mode of evaluation), the attending faculty stepped back into the simulation area and introduced the equipment that had been hidden from direct student view: the bedside ultrasound machine with a transvaginal probe. The ultrasound portion of the simulation included real-time ultrasound interpretation revealing normal anatomy on the left and a tubo-ovarian abscess on the right. As students manipulated the vaginal ultrasound probe, faculty guided the visualization of important anatomic features including the bladder, uterus and endometrium, fallopian tubes, ovaries, and retroperitoneal space. Thirty minutes were allotted for each small group doing the simulation case. As many students as possible within the allotted time were allowed to use the transvaginal ultrasound probe to visualize the accompanying normal and abnormal pathology.

Copyright © 2021 Pearson et al. This is an open-access publication distributed under the terms of the Creative Commons Attribution license.

Debriefing
After both groups within the learning community had completed the simulation, a 30-40 minute debrief was done with the entire learning community. This debrief incorporated further self-evaluation, faculty feedback, and small-group discussion. The debrief was scheduled in a small-group classroom apart from the simulation room, allowing accommodation for the entire learning community. This schedule also allowed for the most efficient use of both the simulation center and required personnel.

The PowerPoint debrief presentation (Appendix B) was used to promote consistent structure of the discussion. This included a discussion of team dynamics as well as specific feedback from the faculty critical action checklist. The debrief included discussion of management options for patients not only with PID, but with complications such as a tubo-ovarian abscess. Discussion included issues such as treatment of sexual partner(s) and potential long-term risks following PID with or without complications such as a tubo-ovarian abscess. As progression through the PowerPoint occurred, any questions that students had in response to the simulation scenario were addressed.

At the end of the debrief session, students were prompted by the slides to fill out the student survey (Appendix D) on this simulation experience, delivered by use of an online Qualtrics survey embedded within the online session assets.

Approximately 7 hours of total curricular time was needed to accommodate the class size of approximately 65 students. Each individual student participated in 60-70 minutes of total curricular activity. During most years of the running of this simulation, all five learning communities completed the scenario and debrief within a single 8-hour day. One year, due to faculty conflicts, the simulation was scheduled over 2 consecutive half-days.

Assessment
The evaluation tools developed for this simulation case included the following: (1) faculty critical action checklist (Appendix C), (2) student survey (Appendix D), and (3) multiple-choice examination questions. The faculty critical action checklist did not require structured data reporting. Rather, this tool provided each small-group faculty the ability to guide both individual and small-group formative assessment and feedback, to assure that educational objectives were met for all students in each small group.

The team of physician faculty facilitating the case—a cohort consisting of family medicine and internal medicine physician faculty with content expertise—collectively developed and modified these tools through the years. The faculty critical action checklist and multiple-choice examination questions were developed according to best practices in management of PID and tubo-ovarian abscess.[14-20] The student survey was developed and vetted for face validity. Modification to assessment tools by these content experts has occurred since case inception. Given changes that occurred year to year, the results being reported were based on the current tools that have been used unchanged since 2018.

Results

Since 2011, approximately 650 students have participated in this simulation case. Five faculty per year facilitated the case and the debrief, as well as contributed to ongoing modifications and updates to the assessment tools and student evaluation.

The student survey distributed for the years 2018-2020 ($N = 93$), provided feedback on students' experience with this simulation. Over all 3 years, all questions had a mean response of 4.5-4.8 on the 5-point scale (1 = *strongly disagree*, 5 = *strongly agree*), demonstrating the consistent agreement by students of the clarity, fidelity, and knowledge-enhancing value of the simulation case and debrief discussion (Table).

The questions at the end of the student survey allowed free-text responses for feedback on the following: (1) the presence or absence of technical problems, (2) what was appreciated about the simulation, (3) what could be improved, and (4) general nondirected comments about their simulation experience. During the 3 years being reported, 86 of the 93 students reported no technical problems. During the year 2018-2019, the cervix fell off of the pelvic model in two small groups, so five of the 41 students reported this occurrence. As for what was valued about this simulation, the main and overwhelmingly consistent theme emerging from students' responses was the ability to incorporate hands-on learning with a pelvic exam, cervical sampling, and a transvaginal ultrasound into this experience. There were no additional consistent themes that emerged from this question.

Table. Student Evaluation of Simulation Experience ($N = 93$)

	M[a]		
**Statement**	**2018**[b]	**2019**[c]	**2020**[d]
Case history was clear.	4.7	4.7	4.4
Simulation was realistic.	4.6	4.6	4.5
Participation in simulation enhanced my knowledge.	4.8	4.7	4.6
Debriefing session was valuable.	4.8	4.7	4.4
Simulation case was valuable.	4.8	4.7	4.5

[a]Rated on a 5-point Likert scale (1 = *strongly disagree*, 5 = *strongly agree*).
[b]$n = 38$
[c]$n = 41$
[d]$n = 14$

Copyright © 2021 Pearson et al. This is an open-access publication distributed under the terms of the Creative Commons Attribution license.

As for improvement suggested, most students commented that they really enjoyed the simulation and that no improvements were needed. From 2018-2020, between two and five students made suggestions for the following: (1) consideration of having the patient's partner in the simulation, (2) desire for more overall time for the simulation, (3) improvements to the simulation mannequin voice/volume, and (4) assuring timely and optimal delivery of lab results within the simulation. The final free-text comment box included many remarks about this being their favorite simulation due to the hands-on learning, as well as the overall value of this simulation in enhancing hands-on skills and clinical thinking.

Multiple-choice examination questions from the simulation and debrief experience further assessed student understanding of the pathophysiology, risk factors, clinical presentation, testing, diagnosis, management, reporting, and complications of PID and tubo-ovarian abscess. Yearly review of student performance on these examination questions within the Hormone and Reproductive Medicine Course assured that the aforementioned knowledge was gained within the simulation experience.

Discussion

The presentation of acute abdominal/pelvic pain in reproductive-aged females is a common occurrence in many medical settings. Thus, teaching medical students how to evaluate pelvic pain in a female patient is a necessary part of undergraduate medical education. Basic examination skills are foundational within the first 2 years of medical school. This case, however, contributed a simulated experience where basic skills were reinforced within a clinical scenario replicating real-life situations students would soon be experiencing on clerkships. The case contributed to students' ability to ask appropriate medical history questions, do an appropriate abdominal and pelvic exam, formulate a differential diagnosis, and work through further evaluation and management issues with a diagnosis such as PID. This workup included beginning skill development for both STI sampling technique and the use of transvaginal ultrasound. This case was unique within the medical literature for the expansive and well integrated educational objectives it has provided within a simulated clinical experience for preclerkship undergraduate medical students. This simulation experience provided a comprehensive foundation that may also help to diminish student anxiety when encountering female patients with pelvic symptoms during clinical clerkships.[9,10]

Unlike some simulation cases where faculty stay completely removed from the case, this simulation allowed for faculty to guide two sections of the students' learning: the pelvic exam that included sampling for a possible STI, as well as a transvaginal ultrasound assessing for possible reproductive tract pathology. Within the context of a clinical scenario, faculty could probe students for their thoughts and/or actions as an attending physician would do in clerkships. As this was the first exposure most students had with the transvaginal ultrasound probe and technique, this simulation experience created an expanded understanding of potential utility and value of this modality of evaluation.

In implementing this simulation, there were some challenges and/or limitations to consider. First, in schools where student numbers make this too time-intensive within the curriculum, a larger group debrief could be considered to diminish overall curricular time. Second, due to the number of students in each small-group simulation, hands-on components of the simulation allowed for only a portion of the students to actually perform the abdominal and female pelvic exams and transvaginal ultrasound during the allotted simulation time. Other students learned by observation and review of the techniques that were discussed by the entire small group. Despite this, and consistent with what others have established,[21] we found that the observing students still reported high value to the experience in reinforcing exam skills and introducing transvaginal ultrasound. One of the ways our school began mitigating this limitation was by allowing students access to the simulation center after the simulation was completed. Students who did not have time for hands-on practice during the simulation were able to practice on the pelvic exam model (with various speculums and sampling equipment included) as well as the ultrasound machine with a transvaginal probe. Faculty oversight and guidance was provided during this optional and unstructured time. Additional time within the simulation scenario itself could also be considered in institutions where curricular scheduling so allows.

In addition, we recognized that the use of multiple pelvic models introduced risk for reducing the fidelity of the experience. The student feedback we have received, however, has supported the fidelity of the experience by our learners. In fact, students have understood the limitations of certain models and, instead of questioning fidelity, have rather expressed appreciation for the opportunity to simulate a complex and sensitive case such as this prior to having to evaluate a similarly presenting real patient.

Another potential challenge was the fact that in real life, a patient presenting with a problem similar to this scenario may be in too much pain to tolerate a transvaginal ultrasound. An abdominal ultrasound probe may be tried first. As the attending faculty

Copyright © 2021 Pearson et al. This is an open-access publication distributed under the terms of the Creative Commons Attribution license.

guided students through this portion of the simulation and the topic of ultrasound evaluation was revisited in the debrief, this issue was discussed. Pros and cons of using an abdominal versus transvaginal probe were discussed, as was the consideration for analgesia in cases where the transvaginal probe was necessary to adequately visualize the underlying pathology. Because this was addressed and discussed, we have found that this potential challenge actually enhanced the educational objective in allowing students to expand their understanding of the value of abdominal versus transvaginal ultrasound evaluation.

Because of the complexity of this simulation in addition to the requirement that basic skills have already been taught, we recognized that additional potential challenges could arise if placed too early in an undergraduate medical school curriculum. We also recognized the budgetary challenge that obtaining expensive equipment may pose if this equipment is not already available. We have run this simulation by using a pelvic model that both did and did not have a purulent cervicitis built into the model. In the scenario without equipment that allowed for a purulent cervicitis, we used a laminated picture of what the examining student would see on a speculum exam (a purulent cervicitis). This did not diminish the fidelity of the experience for students when compared with other years, and in fact allowed other students not performing the exam to easily visualize the findings as well. Also, in institutions without a pelvic model showing the underlying tubo-ovarian abscess pathology and/or where concern for cost of this equipment could be limiting, this complication could be taken out of the simulation. In this modification, due to the severity of the presenting patient, transvaginal ultrasound could be introduced in an effort to rule out underlying pathology, and the findings could simply reinforce normal underlying anatomy. This modified scenario could be run with one pelvic model on which both the pelvic exam and transvaginal ultrasound could be performed.

Lastly, we recognized that the tools we have provided for measuring the educational effectiveness of this simulation have limitations in being able to provide reproducible and clearly structured data. The size of our overall student population as well as the consistent teaching by individual faculty within a given learning community over 2 years have allowed for a level of consistent formative evaluation and feedback that may not be feasible in some schools. Thus, the development of more structured evaluation tools may be desired in some institutions.

This case has generalizability to any undergraduate medical school desiring to create a simulated opportunity in which to accomplish the associated learning objectives. At our school, we intend to continue to provide this simulation experience for our second-year medical students within the Hormone and Reproductive Medicine Course prior to the transition into clerkships. Faculty evaluation and student feedback have consistently confirmed that this simulation provided a valuable, appreciated, and knowledge-enhancing experience.

Appendices

A. Simulation Case.docx

B. Debrief PowerPoint.pptx

C. Faculty Critical Action Checklist.docx

D. Student Survey.docx

All appendices are peer reviewed as integral parts of the Original Publication.

Jennifer Pearson, MD: Assistant Professor, Department of Family Medicine and Biobehavioral Health, University of Minnesota Duluth; ORCID: https://orcid.org/0000-0002-4289-3893

Amy Greminger, MD: Assistant Professor, Department of Family Medicine and Biobehavioral Health, University of Minnesota Duluth

Emily Onello, MD: Assistant Professor, Department of Family Medicine and Biobehavioral Health, University of Minnesota Duluth

Sandy Stover, MD: Assistant Professor, Department of Family Medicine and Biobehavioral Health, University of Minnesota Duluth

Disclosures
None to report.

Funding/Support
None to report.

Ethical Approval
Reported as not applicable.

References

1. Stratton P. Evaluation of acute pelvic pain in non-pregnant adult women. UpToDate. Updated May 9, 2019. Accessed June 23, 2020. https://www.uptodate.com/contents/evaluation-of-acute-elvic-pain-in-nonpregnant-adult-women?search=evaluation%20of%20acute%20pelvic%20pain%20in%20women&source=search_result&selectedTitle=1~57&usage_type=default&display_rank=1
2. Jennings LK, Krywko DM. Pelvic inflammatory disease. In: *StatPearls*. StatPearls Publishing; 2020. https://www.ncbi.nlm.nih.gov/books/NBK499959/

Copyright © 2021 Pearson et al. This is an open-access publication distributed under the terms of the Creative Commons Attribution license.

3. Patel MD, Young SW, Dahiya N. Ultrasound of pelvic pain in the nonpregnant woman. *Radiol Clin North Am*. 2019;57(3):601-616. https://doi.org/10.1016/j.rcl.2019.01.010
4. Powell HS, Bridge J, Eskesen S, Estrada F, Laya M. Medical students' self-reported experiences performing pelvic, breast, and male genital examinations and the influence of student gender and physician supervision. *Acad Med*. 2006;81(3): 286-289. https://doi.org/10.1097/00001888-200603000-00022
5. Peled J, Thompson D, Trentacoste S, Katz N. Female breast and pelvic exam: a student-to-student guide. *MedEdPORTAL*. 2009;5:1653. https://doi.org/10.15766/mep_2374-8265.1653
6. Posner G. "Sensitive" examination workshop for medical clerkship students. *MedEdPORTAL*. 2010;6:7936. https://doi.org/10.15766/mep_2374-8265.7936
7. Nelson EL. Teaching the female breast and pelvic exam: a student- and patient-centered approach. *MedEdPORTAL*. 2016; 12:10399. https://doi.org/10.15766/mep_2374-8265.10399
8. Dugoff L, Pradhan A, Casey P, et al. Pelvic and breast examination skills curricula in the United States medical schools: a survey of obstetrics and gynecology clerkship directors. *BMC Med Educ*. 2016;16(1):314. https://doi.org/10.1186/s12909-016-0835-6
9. Hugon-Rodin J, Sonigo C, Drummond D, et al. Learning the gynecological examination on low-fidelity simulation: impact on the feelings of medical students. *Gynecol Obstet Fertil Senol*. 2017;45(5):291-298. https://doi.org/10.1016/j.gofs.2017.03.011
10. Dabson AM, Magin PJ, Heading G, Pond D. Medical students' experiences learning intimate physical examination skills: a qualitative study. *BMC Med Educ*. 2014;14:39. https://doi.org/10.1186/1472-6920-14-39
11. Task Force on the Clinical Skills Education of Medical Students. *Recommendations for Preclerkship Clinical Skills Education for Undergraduate Medical Education*. Association of American Medical Colleges. 2008. Accessed December 5, 2020. https://www.stfm.org/media/1363/clinicalskills_oct09qxdpdf.pdf
12. Clifton M. Adolescent pelvic inflammatory disease problem based learning (PBL). *MedEdPORTAL*. 2007;3:264. https://doi.org/10.15766/mep_2374-8265.264
13. Davis J, Weinberger L. Emergency medicine simulation scenario: first-trimester "septic" abortion. *MedEdPORTAL*. 2011;7:9027. https://doi.org/10.15766/mep_2374-8265.9027
14. Beigi RH. Management and complications of tubo-ovarian abscess. UpToDate. Updated May 22, 2020. Accessed June 23, 2020. https://www.uptodate.com/contents/management-and-complications-of-tubo-ovarian-abscess?search=managment%20of%20tuboovarian%20abscess&source=search_result&selectedTitle=1~24&usage_type=default&display_rank=1
15. Curry A, Williams T, Penny ML. Pelvic inflammatory disease: diagnosis, management, and prevention. *Am Fam Physician*. 2019;100(6):357-364.
16. *Sexually Transmitted Diseases (STDs).* Minnesota Department of Health. Updated January 31, 2019. Accessed December 5, 2020. https://www.health.state.mn.us/diseases/stds/index.html
17. Farid H, Karmon EA, Styer AK. Inpatient management of tubo-ovarian abscesses: what is the threshold of parenteral antibiotic treatment failure? *Obstet Gynecol*. 2015;125(suppl 1):23S. https://doi.org/10.1097/01.AOG.0000465329.80185.3a
18. Rosen M, Breitkopf D, Waud K. Tubo-ovarian abscess management options for women who desire fertility. *Obstet Gynecol Surv*. 2009;64(10):681-689. https://doi.org/10.1097/OGX.0b013e3181b8b0d6
19. *2015 Sexually Transmitted Diseases Treatment Guidelines*. Centers for Disease Control and Prevention. 2015. Accessed December 5, 2020. https://www.cdc.gov/std/tg2015/default.htm
20. *Final Recommendation Statement: Chlamydia and Gonorrhea: Screening*. US Preventive Services Task Force. September 22, 2014. Accessed December 5, 2020. https://www.uspreventiveservicestaskforce.org/uspstf/document/RecommendationStatementFinal/chlamydia-and-gonorrhea-screening
21. Kaplan BG, Abraham C, Gary R. Effects of participation vs. observation of a simulation experience on testing outcomes: implications for logistical planning for a school of nursing. *Int J Nurs Educ Scholarsh*. 2012;9(1):14. https://doi.org/10.1515/1548-923X.2398

Received: July 14, 2020
Accepted: October 8, 2020
Published: January 25, 2021

Copyright © 2021 Pearson et al. This is an open-access publication distributed under the terms of the Creative Commons Attribution license.
